# Transcriptomic analysis of the dialogue between Pseudorabies virus and porcine epithelial cells during infection

**DOI:** 10.1186/1471-2164-9-123

**Published:** 2008-03-10

**Authors:** Laurence Flori, Claire Rogel-Gaillard, Marielle Cochet, Gaetan Lemonnier, Karine Hugot, Patrick Chardon, Stéphane Robin, François Lefèvre

**Affiliations:** 1 INRA, DGA, UMR 314, Laboratoire de Radiobiologie et d'Etude du Génome, Jouy-en-Josas, F-78350 France; CEA, DSV, IRCM, SREIT, Laboratoire de Radiobiologie et d'Etude du Génome, Jouy-en-Josas, F-78350, France; 2 INRA, DSA, UR892, Unité de Virologie et Immunologie Moléculaires, Jouy-en-Josas, F-78350, France; 3 AgroParisTech-ENGREF-INRA, UMR 518, Unité de Mathématiques et Informatique Appliquées, Paris F-75005, France

## Abstract

**Background:**

Transcriptomic approaches are relevant for studying virus-host cell dialogues to better understand the physiopathology of infection and the immune response at the cellular level. Pseudorabies virus (PrV), a porcine Alphaherpesvirus, is a good model for such studies in pig. Since PrV displays a strong tropism for mucous epithelial cells, we developed a kinetics study of PrV infection in the porcine PK15 epithelial cell line. To identify as completely as possible, viral and cellular genes regulated during infection, we simultaneously analyzed PrV and cellular transcriptome modifications using two microarrays i.e. a laboratory-made combined SLA/PrV microarray, consisting of probes for all PrV genes and for porcine genes contained in the Swine Leukocyte Antigen (SLA) complex, and the porcine generic Qiagen-NRSP8 oligonucleotide microarray. We confirmed the differential expression of a selected set of genes by qRT-PCR and flow cytometry.

**Results:**

An increase in the number of differentially expressed cellular genes and PrV genes especially from 4 h post-infection (pi) was observed concomitantly with the onset of viral progeny while no early global cellular shutoff was recorded. Many cellular genes were down-regulated from 4 h pi and their number increased until 12 h pi. UL41 transcripts encoding the virion host shutoff protein were first detected as differentially expressed at 8 h pi. The viral gene UL49.5 encoding a TAP inhibitor protein was differentially expressed as soon as 2 h pi, indicating that viral evasion via TAP inhibition may start earlier than the cellular gene shutoff. We found that many biological processes are altered during PrV infection. Indeed, several genes involved in the SLA class I antigenic presentation pathway (SLA-Ia, TAP1, TAP2, PSMB8 and PSMB9), were down-regulated, thus contributing to viral immune escape from this pathway and other genes involved in apoptosis, nucleic acid metabolism, cytoskeleton signaling as well as interferon-mediated antiviral response were also modulated during PrV infection.

**Conclusion:**

Our results show that the gene expression of both PrV and porcine cells can be analyzed simultaneously with microarrays, providing a chronology of PrV gene transcription, which has never been described before, and a global picture of transcription with a direct temporal link between viral and host gene expression.

## Background

*In vitro *analyses of host cell/pathogen interactions are essential to unravel the mechanisms of infection and to investigate the host response to infection. Pseudorabies virus (PrV) belongs to the *Alphaherpesvirinae *subfamily as for example the human herpes simplex virus 1 (HSV-1) and is a well-known pig pathogen responsible for Aujeszky's disease, causing considerable economical losses worldwide in this species [1]. Although some countries have succeeded in eradicating Aujeszky's disease through vaccination and health policies, the disease prevalence still remains variable in other countries. Young piglets are more severely affected by PrV infection often resulting in fatal encephalitis, than older infected pigs, which can remain asymptomatic or develop mild to severe respiratory disease symptoms associated with a limited mortality. Indeed, PrV displays a strong tropism for epithelial cells of the oronasal respiratory tract, which are the first cells targeted by virions [1,2]. Abortions, stillbirths or weak piglets that die within 48 h of birth are also observed when pregnant sows are infected [1]. Moreover, PrV can infect a broad range of vertebrates resulting in a uniform lethality but it is generally considered as a non-pathogenic agent for man [3]. Because PrV is easy to propagate in cells of several mammalian species including rodents and is not harmful to laboratory workers, PrV is a highly relevant model to study the biology of alphaherpesviruses and their interactions with host cells *in vitro *[1]. In addition, its genome has been reconstructed from sequences of six different strains (Kaplan, Becker, Rice, Indiana-Funkhauser, NIA-3, and TNL) and 70 genes encoding structural and non structural proteins have been annotated [4].

Viruses have evolved strategies to evade the host immune response. In particular, herpesviruses interfere with the Major Histocompatibility Complex (MHC) class I antigen presentation pathway to avoid the Cytotoxic T Lymphocyte (CTL) response [5]. MHC class I molecules are expressed on almost all nucleated cells and present peptides, including peptides derived from viral antigens, to CTL, which play a critical role in the defense mechanisms against viral infection. It has been reported that PrV infection decreases the expression of MHC class I molecules on the cell surface [6]. This down-regulation is partly explained by the inhibition of the ABC transporter TAP activity, due to interactions with the viral gN protein encoded by the UL49.5 gene [7,8]. This inhibition is independent of the non-specific mRNA cellular shut-off produced by the virion host shut-off (vhs) protein encoded by the UL41 gene [9]. However, mechanisms other than TAP inhibition may be involved in avoiding the MHC class I presentation pathway.

A precise and more complete identification of cellular and viral genes, which are up- or down-regulated during the time course of infection, is essential to better understand the physiopathology of infection and to identify the molecules involved in host resistance/susceptibility mechanisms. During recent years, DNA microarray technology has proven to be a very efficient high-throughput tool to study the gene expression profiles of infected host cells or pathogens [10,11]. To date, three transcriptomic analyses focused on cellular gene expression have been carried out in non-porcine PrV infected cells [12-14]. Ray and Enquist have compared the cellular pathways regulated by PrV and HSV-1 during infection of rat embryonic fibroblast cells using a rat microarray [12]. In a similar system, Brukman and Enquist have explored how PrV evades the IFN-mediated immune response [13]. Finally, Blanchard *et al *have used a human microarray to characterize the impact of PrV infection in human embryonic kidney cells (HEK-293) [14]. These studies have identified many biological processes and host cell genes regulated during infection. The next step in a transcriptomic approach would be the simultaneous analysis of viral and cellular modifications of transcription during PrV infection [10,11] using porcine genomic tools. Since the pig whole genome assembly is not yet achieved, no complete pan-genomic array exists and only partial generic microarrays are commercially available [15]. However, the pig MHC region referred to as the SLA (Swine Leukocyte Antigen) complex, located on chromosome 7, is the first region of the pig genome that is entirely sequenced and annotated [16].

In this context, our aim was to study the dialogue between PrV and the PK15 porcine epithelial cell line, which mimics the first porcine target cells. Using two different porcine microarrays, we followed both the viral and cellular transcriptome kinetics during infection. These microarrays were the Qiagen-NRSP8 commercial array [17] and a microarray we constructed, referred to as SLA/PrV, which combines probe sets specific to genes localized in the SLA complex, genes encoding other important immunological molecules [18] and all the PrV genes. Here, we present a large-scale analysis of the porcine physiological pathways regulated during viral infection with a special focus on genes in the SLA complex together with the modifications of the PrV transcriptome.

## Results

### Construction of the SLA/PrV microarray and complementarity with the Qiagen-NRSP8 microarray

**Figure 1:**
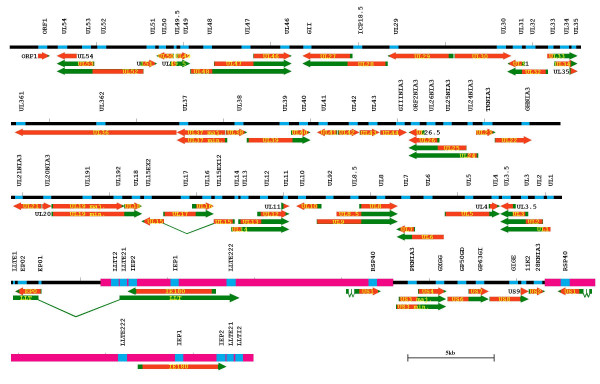
**Map of the PrV transcriptome. **The relative location of transcripts and designed amplicons are shown. UL and US regions of the PrV genome are represented in black while IR and TR regions are in pink. Transcripts corresponding to each gene are represented with arrows (coding region in orange and non-coding regions or introns in green). Amplicons are represented in blue on the genome. The name of each amplicon is written above the corresponding genome location. Size is measured in kb.

The 1789 DNA/cDNA probes spotted on the SLA/PrV microarray fall into four distinct probe sets: i) 420 probes localized on a segment of chromosome 7 between the loci PRL and PRIM2A (SSC7p1.1-q1.2), which includes the extended-SLA region and represents 272 unique sequences, 111 belonging to the strict SLA region between the loci UBD and RING1 [16]; ii) 73 probes specific to 73 genes encoding molecules involved in immunity and localized outside the SLA region [18]; iii) 80 PrV probes specific to the 70 viral genes (Figure 1) and iv) 1170 probes randomly chosen for data normalization from porcine cDNA AGENAE library [19,20]. The PrV/SLA microarray covers 72.5% of the annotated sequences of the strict SLA region (= 111/153) [16,21].

The Qiagen-NRSP8 oligonucleotide microarray contains 13297 probes, which match 8541 unique human or mouse RefSeq or pig annotated gene NCBI accession numbers and 1.5% of these encode immune proteins [17]. Only 48 of the 420 probes from the extended SLA probe set and 41 of the 73 from the immune probe set are present on both microarrays.

### Expression of PrV genes during the time course of infection

**Figure 2:**
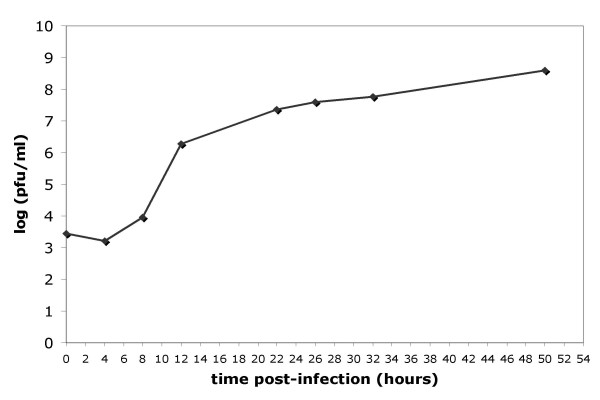
**PrV growth kinetics in PK15 cells. **The PK15 cells were infected with PrV NIA3 at 20 MOI in HMS-M medium. PrV was titrated in the medium by plaque assay at different times pi (0, 4, 8, 12, 22, 26, 32 and 50 h pi). PrV titer was expressed as plaque forming units per ml (pfu/ml).

**Figure 3:**
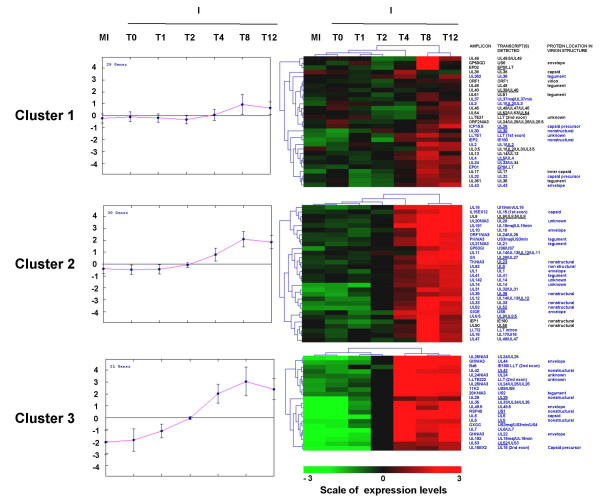
**Clusters of PrV gene expression levels identified by the k-means method. **The average of normalized intensities for all mock-infected conditions and for each infected condition, centered (median) by genes were analyzed with the k-means method (three clusters). For each cluster, one graph and one clustering picture are represented. The graph shows the mean of the expression levels of all genes (x = Time; y = mean of levels of expression). The clustering picture depicts the mean of each gene expression level for all mock-infected time points and for each infected time point (x = Time; y = level of expression). The list of PrV amplicons belonging to each cluster, the list of the corresponding viral genes and the location of the corresponding proteins in virion structure (for amplicons that hybridize to a single transcript) are represented on the right. The names of the probes that are differentially expressed are represented in blue. To distinguish immediate early, early and late genes, early genes are underlined. All other genes are late genes except IE180 which is the only immediate early gene of PrV.

The six time points, which were studied in this experiment – i.e. 0, 1, 2, 4, 8 and 12 hours (h) post-infection (pi), were chosen according to viral growth kinetics observed in PK15 cells in our experimental conditions (Figure 2). The expression of viral genes was detected between 2 and 12 h pi and increased during time and most of the genes were expressed at 8 and 12 h pi. The hierarchical clustering (HCL) of viral gene expression levels according to all conditions (time and infection status) allowed us to distinguish two main groups: i) mock-infection at all time points and infection until 2 h pi ii) infection from 4 until 12 h (data not shown). With the k-means method, we identified three transcript clusters with similar expression profiles (Figure 3). The average expression levels for the first cluster (29 probes) showed little variation and only from 8 h pi. The second cluster contained 30 probes corresponding to genes, the expression level of which increased from 4 h pi. The last group (21 probes) displayed a higher increase of expression level from 2 to 8 h pi.

Using a linear model and student t tests, the analysis of viral genes, which were differentially expressed between infected and mock-infected cells at each time pi (FDR = 0.05) indicated that three differentially expressed probes were observed as soon as 1 h pi and that this number increased drastically between 2 and 8 h (39 additional probes) and reached a plateau after 8 h pi (Table 1). The important increase between 4 and 8 h confirmed the HCL and k-means results and correlated with the PrV growth curve (Figure 2). Nineteen probes were not differentially expressed between infected and mock-infected cells at any time and 16 of them were found in the less variable cluster by the k-means method (Figure 3). Most of the differentially expressed probes belonged to the two most variable clusters. Among the 58 differentially expressed probes, 26 recognized two to four transcripts and 32 were specific to a single transcript (Table 2). When examining these latter 32 genes, the first differentially expressed genes observed 1 h pi were US1 and UL29 encoding two non structural proteins RSP40 and ICP8, respectively, and UL49.5 encoding the virion envelope glycoprotein gN. However, IE180 encoding the immediate early protein IEP, a transactivator of early gene expression was found differentially expressed at 4 h pi. Moreover, the four genes, which were differentially expressed between 8 and 12 h, specify non structural (UL9), capsid (UL28), tegument (UL36) and envelope (US8) transcripts. The differential expression of UL41 encoding vhs protein was first detected at 8 h pi. Among the probes recognizing two transcripts, PKNIA3 specific of US3min and US3maj involved in the inhibition of apoptosis was also differentially expressed from 8 h pi and UL37 specific of UL37min and UL37maj encoding a tegument protein was differentially expressed at 12 h pi.

**Table 1:** Number of viral and cellular probes differentially expressed at each time point.

Probe set	Microarray	Probe subset	T0	T1	T2	T4	T8	T12
PrV	SLA/PrV	Probes homologous to a single transcript	0	3	7	18	26	30
		Total	0	3	13	34	52	58

Pig	SLA/PrV	up-regulated SLA/Immune probes	0	0	0	1	4	3
		down regulated SLA/Immune probes	0	0	0	11	18	13
		up-regulated random probes	1	0	2	4	15	11
		down-regulated random probes	0	0	0	20	70	80
		Total	1	0	2	36	107	107
	
	Qiagen- NRSP8	up-regulated probes	ND*	27	134	1262	3184	ND*
		down-regulated probes	ND*	4	47	1494	3509	ND*
		Total	ND*	31	181	2756	6693	ND*

*ND: not determined

**Table 2:** Viral probes and fold-change during infection kinetics.

Amplicon name	Transcript name	Fold-change						Encoded protein and function*	Structural role*
				
		T0	T1	T2	T4	T8	T12		
ORF1	ORF1	-	-	-	-	-		unknown	virion
UL54	UL52/UL53/UL54	-	-	-	-	-	-		
UL53	UL52/UL53	-	-	4.6	6.6	6.7	7.4		
UL52	UL52	-	-	-	4.7	6.4	5.6	DNA replication; primase subunit of UL5/UL8/UL52complex	nonstructural
UL51	UL51	-	-	-	-	-	-	viral egress	tegument
UL50	UL50	-	-	-	-	-	-	dUTPase	nonstructural
UL49.5	UL49.5	-	4.4	6.4	9.0	10.4	13.5	gN; immune evasion (TAP inhibitor)	envelope
UL49	UL49.5/UL49	-	-	-	-	-	-		
UL48	UL48	-	-	-	-	-	-	VP16, α-TIF; gene regulation(transactivator); viral egress	tegument
UL47	UL48/UL47	-	-	-	-	4.0	3.9		
UL46	UL48/UL47/UL46	-	-	-	-	-	-	VP11/12; unknown; tegument protein	
GII	UL28/UL27	-	-	-	-	6.7	6.3		
ICP18.5	UL28	-	-	-	-	-	7.4	ICP18,5; DNA cleavage and packaging; component of the UL15/UL28 terminase	capsid precursor
UL29	UL29	-	6.0	9.2	10.6	8.9	10.9	ICP8; DNA replication and recombination; binds ssDNA	nonstructural
UL30	UL30	-	-	4.2	-	-	-	DNA replication; DNA polymerase subunit of UL30/UL42 holoenzyme	nonstructural
UL31	UL32/UL31	-	-	-	4.5	9.2	10.1		
UL32	UL32	-	-	-	-	4.0	4.1	DNA packaging; efficient localization of capsids to replication compartments	capsid precursor
UL33	UL33	-	-	-	-	4.5	5.8	DNA cleavage and packaging; associates with UL28 and UL15	nonstructural
UL34	UL33/UL34	-	-	-	-	6.1	5.0		
UL35	UL33/UL34/UL35	-	-	5.6	7.1	9.5	9.5		
UL361	UL36	-	-	-	-	-	-	VP1/2; viral egress(capsid tegumentation); interacts with Ul37 and capsid	tegument
UL362	UL36	-	-	-	-	-	3.8	VP1/2; viral egress(capsid tegumentation); interacts with Ul37 and capsid	tegument
UL37	UL37maj/UL37min	-	-	-	-	-	5.0		
UL38	UL38	-	-	-	-	-	-	VP19c; minor capsid protein; UL38/UL18/UL18 triplex component	capsid
UL39	UL39	-	-	-	-	4.4	6.3	nucleotide synthesis; large subunit of ribonucleotide reductase	nonstructural
UL40	UL39/UL40	-	-	-	-	-	-		
UL41	UL41	-	-	-	-	4.2	6.8	VHS, gene regulation; Rnase, degrades host and viral mRNAs	tegument
UL42	UL42	-	-	-	6.3	6.9	4.6	DNA replication; polymerase accessory subunit of UL30/UL42 holoenzyme	nonstructural
UL43	UL43	-	-	-	-	4.1	6.2	inhibits glycoprotein-mediated membrane fusion; type III membrane protein	envelope
GIIINIA3	UL44	-	-	-	8.2	12.7	13.9	gC; viral entry (virion attachment); type I membrane protein	envelope
ORF2NIA3	UL24/UL25/UL26/UL26.5	-	-	-	-	-	-		
UL26NIA3	UL24/UL25/UL26	-	-	-	6.7	8.3	7.6		
ORF1NIA3	UL24/UL25					8.1	8.0		
UL25NIA3	UL24/UL25	-	-	-	8.3	10.7	12.2		
UL24NIA3	UL24	-	-	-	6.9	9.8	10.4	unknown, type III membrane protein	unknown
TKNIA3	UL23	-	-	-	-	5.0	6.8	TK, nucleotide synthesis; thymidine kinase; selectively activates acyclovir	nonstructural
GHNIA3	UL22	-	-	4.8	6.0	8.8	9.0	gH; viral entry (fusion); cell-cell spread; type i membrane PT	envelope
UL21NIA3	UL21	-	-	-	-	5.9	5.5	unknown; capsid-associated tegument protein; interacts with UL16	tegument
UL20NIA3	UL20	-	-	-	3.8	5.3	7.3	viral egress, type III membrane protein	unknown
UL191	UL19maj/UL19min	-	-	-	5.0	9.7	10.8		
UL192	UL19maj/UL19min	-	-	6.6	8.6	12.6	12.9		
UL18	Ul19min/UL18	-	-	-	7.0	5.7	9.6		
UL15EX2	UL15 (2^nd ^exon)	-	-	6.4	6.5	7.5	9.2	DNA cleavage/encapsidation; terminase subunit of UL15/UL28 terminase6	capsid precursor
UL17	UL17	-	-	-	-	-	-	DNA cleavage and encapsidation	inner capsid
UL16	UL17/Ul16	-	-	-	4.5	6.2	5.0		
UL15EX12	UL15 (1^st ^exon)	-	-	-	-	4.0	7.7	DNA cleavage/encapsidation; terminase subunit of UL15/UL28 terminaseUL6	capsid precursor
UL14	UL14				6.1	6.1	9.8	unknown	unknown
UL142	UL14	-	-	-	-	6.3	7.8	unknown	unknown
UL13	UL14/UL13	-	-	-	-	-	-		
UL12	UL14/UL13/UL12	-	-	-	-	3.7	5.3		
UL11	UL14/UL13/UL12/UL11	-	-	-	-	-	6.0		
UL10	UL10	-	-	-	-	6.6	6.1	gM; inhibits glycoprotein-mediated membrane fusion; type III membrane protein	envelope
UL92	UL9	-	-	-	-	-	4.1	not found	non structural
UL8.5	UL9/UL8.5	-	-	-	4.0	-	-		
UL8	UL9/UL8.5/UL8	-	-	-	-	-	-		
UL7	UL6/UL7	-	-	5.0	7.4	10.7	9.4		
UL6	UL6	-	-	5.0	12.7	13.8	13.3	capsid protein; portal protein; docking site for terminase	capsid
UL5	UL5	-	-	4.3	6.3	8.7	5.9	DNA replication; part of UL5/UL8/UL52 helicase/primase complex	nonstructural
UL4	UL5/UL4	-	-	-	-	3.5	5.5	unknown	
UL3.5	UL1/UL2/UL3/UL3.5	-	-	-	-	-	-		
UL3	UL1/UL2/UL3	-	-	-	-	5.1	6.5		
UL2	UL1/UL2	-	-	-	4.2	5.4	5.4		
UL1	UL1	-	-	-	-	6.5	7.3	gL; viral entry (fusion); sell-sell spread	envelope
LLTE1	LLT (1^st ^exon)	-	-	-	4.6	6.5	8.0	not found	unknown
EP02	EP0/LLT	-	-	-	-	-	-		
EP01	EP0/LLT	-	-	-	-	6.3	4.7		
LLTI2	LLT intron	-	-	-	-	3.7	4.7		
LLTE21	LLT (2^nd ^exon)	-	-	-	-	-	-	not found	unknown
IEP2	IE180	-	-	-	3.5	-	-	ICP4; gene regulation (transactivator); immediate early protein	nonstructural
IEP1	IE180	-	-	-	-	-	-	ICP4; gene regulation (transactivator); immediate early protein	nonstructural
Ba5	IE180/LLT (2^nd ^exon)				5.9	6.7	4.3		
LLTE222	LLT (2^nd ^exon)	-	-	-	6.8	8.9	8.2	not found	unknown
RSP40	US1	-	5.3	8.3	12.9	15.3	13.0	RSP40/ICP22; unknown; HIV-1 homolog acts as regulator of gene expression	nonstructural
PKNIA3	US3maj/US3min	-	-	-	-	6.0	5.0	PK, minor and major form of protein kinase; inhibits apoptosis	tegument
GXGG	US3maj/US3min/US4	-	-	6.5	9.1	10.6	11.0		
GP50GD	US6	-	-	-	-	-	-	gD; viral entry, type I membrane protein	envelope
GP63GI	US6/US7	-	-	-	5.0	6.3	8.5		
GIGE	US8	-	-	-	4.0	-	4.3	gE; cell-cell spread	envelope
11K2	US9/US8	-	-	-	9.0	10.0	10.4		
28KNIA3	US2	-	-	-	6.2	6.9	7.1	28K, tegument protein; membrane associated protein	tegument

*gene function and structural role [1] are only specified for amplicons that hybridize to a single transcript.

- gene not found differentially expressed between PrV infected and mock-infected cells

### Global PK15 differential gene expression during the time course of infection

**Figure 4:**
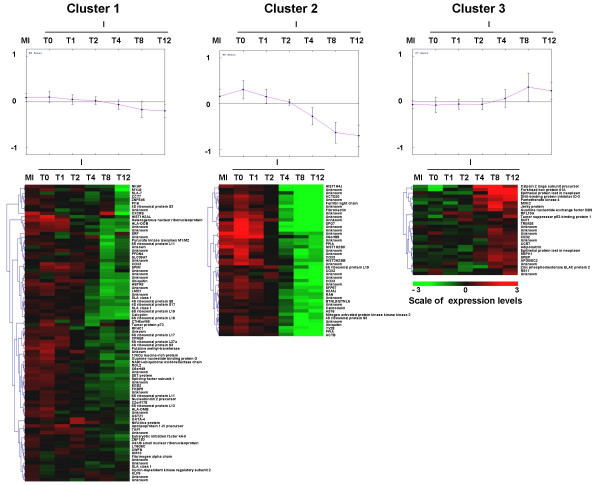
**Clusters of PK15 gene expression levels identified by the k-means method. **The expression levels of genes that are differentially expressed between infected and mock-infected cells at each time point are analyzed by the k-means method (three clusters). Averages of normalized intensities for all mock-infected conditions and for each infected condition were centered (median) by genes. For each cluster, one graph and one clustering picture are represented. The graph shows the mean of the expression levels of all genes (x = Time; y = mean of levels of expression) and the clustering picture depicts the mean of each gene expression level for all mock-infected time points and for each infected time point (x = Time; y = level of expression).

The number of differentially expressed cellular probes increased with time in parallel to viral gene expression (Table 1). Between 0 and 2 h pi most of the SLA/immune probes showed no change and few differentially expressed genes were detected from 1 h pi with the Qiagen-NRPS8 microarray (Table 1). As shown in Table 1, the SLA/PrV microarray identified 1, 0, 2, 36, 107 and 107 differentially expressed probes at 0, 1, 2, 4, 8 and 12 h pi, respectively [see Additional file 1] and the Qiagen-NRSP8 microarray identified 31, 181, 2756, 6693 differentially expressed probes at 1, 2, 4 and 8 h pi [see Additional file 2], respectively. The SLA/PrV microarray data show that 86 (31/36), 82 (88/107), and 87 % (93/107) of the differentially expressed probes were down-regulated at 4, 8 and 12 h pi, respectively and the Qiagen-NRSP8 microarray data show that 13 (4/31), 26 (47/181), 54 (1494/2756) and 52 % (3509/6693) of the differentially expressed probes explored in this case were down-regulated at 1, 2, 4 and 8 h pi, respectively (Table 1). With the k-means method, the expression levels for each condition (time and infection status) of the SLA/PrV differentially expressed probes set were clustered in three groups (Figure 4). Eighty-eight probes with a small decrease in expression levels from 4 h pi were found in the first cluster and 45 probes with a stronger decrease in expression levels from 2 h pi in the second cluster. The third cluster contained 27 up-regulated probes at 8 h pi. The results obtained with both microarrays show that many cellular genes were down-regulated during the time course of the experiment especially between 4 and 12 h pi.

### PrV infection alters multiple biological processes and cellular functions

For each time point, the differentially expressed genes from the Qiagen-NRSP8 microarray were classified into biological processes using GO terms when available (Table 3). The biological processes that contained more than 5% of the differentially expressed genes during the period between 2 and 8 h pi included: protein metabolism and modification (BP0060), nucleoside, nucleotide and nucleic acid metabolism (BP00031), developmental process (BP00193), signal transduction (BP00102), transport (BP00141), cell cycle (BP00203), immunity and defense (BP00148), intracellular protein traffic (BP00125) and cell structure and motility (BP00285). Several biological processes were predominantly regulated 1 h pi such as developmental processes (BP00193) and signal transduction (BP00102). Other biological processes were regulated later such as cell adhesion (BP00124) or apoptosis (BP00179) from 2 h pi and homeostasis (BP00267) from 4 h pi.

**Table 3:** Biological processes associated with differentially expressed cellular genes.

Biological process*	Time pi (h)^§^			
	T1	T2	T4	T8
Protein metabolism and modification (BP00060)	1	26	311	768
Nucleoside, nucleotide and nucleic acid metabolism (BP00031)	3	22	321	702
Developmental processes (BP00193)	8	16	224	487
Signal transduction (BP00102)	5	14	337	737
Transport (BP00141)	1	13	139	308
Cell cycle (BP00203)	2	12	124	262
Immunity and defense (BP00148)	1	11	120	309
Intracellular protein traffic (BP00125)	2	10	115	266
Cell structure and motility (BP00285)	2	7	119	279
Cell proliferation and differentiation (BP00224)	2	6	100	215
Other metabolism (BP00289)	2	6	71	159
Apoptosis (BP00179)	0	6	61	132
Cell adhesion (BP00124)	0	5	68	136
Carbohydrate metabolism (BP00001)	0	3	67	165
Amino acid metabolism (BP00013)	1	3	36	70
Protein targeting and localization (BP00137)	1	3	25	65
Blood circulation and gas exchange (BP00209)	0	3	9	26
Oncogenesis (BP00281)	0	2	57	116
Neuronal activities (BP00166)	1	2	50	106
Lipid, fatty acid and steroid metabolism (BP00019)	0	1	71	183
Electron transport (BP00076)	0	1	32	69
Muscle contraction (BP00173)	0	1	26	55
Sensory perception (BP00182)	0	1	25	60
Coenzyme and prosthetic group metabolism (BP00081)	0	1	19	46
Miscellaneous (BP00211)	0	1	12	30
Phosphate metabolism (BP00095)	0	1	8	27
Sulfur metabolism (BP00101)	0	1	8	22
Nitrogen metabolism (BP00090)	0	1	5	5
Non-vertebrate process (BP00301)	0	0	1	3
Homeostasis (BP00267)	0	0	16	49
Biological process unclassified (BP00216)	7	40	549	1232

Number of analyzed genes	24	141	1272	4458

^§ ^The number of genes from the Qiagen_NRSP8 microarray found in Panther database is reported at each time point for each biological process.

*The same focus gene may exist in different biological processes.

The Ingenuity Pathway Analysis (IPA) of the differentially expressed probes from the Qiagen-NRSP8 microarray identified 82 different top functions associated with significant networks (Table 4). Three top functions were regulated early during infection (1 h pi): gene expression, molecular transport and drug metabolism. Sixteen, 68 and 67 top functions were modulated by PrV infection at 2, 4 and 8 h pi. Fifteen and 14 top functions were specific of time points 4 and 8 h pi, respectively. The number of regulated top functions strongly increased from 4 h pi. The top functions containing the highest number of focus genes at both 4 and 8 h pi were those involved in cancer, cell cycle and cell signaling with the first two detected as early as 2 h pi. Immune response and immunological disease top functions were found from 4 h pi and immune and lymphatic system development and function at 8 h pi. Cell death top function was first detected at 2 h pi.

**Table 4:** Top functions associated with significant networks identified by IPA and number of focus genes at each time point.

Top functions	Time			
	T1	T2	T4	T8
Gene Expression	9	12	178	297
Molecular Transport	9	-	81	372
Drug Metabolism	9	-	31	83
Cancer	-	47	386	357
Connective Tissue Development and Function	-	27	103	152
Cell Cycle	-	24	246	432
Cellular Development	-	23	144	64
Cell Death	-	23	141	205
Cell Morphology	-	23	91	58
Cellular Movement	-	15	92	125
Cellular Assembly and Organization	-	15	82	358
Post-Translational Modification	-	13	75	79
Small Molecule Biochemistry	-	13	64	324
Amino Acid Metabolism	-	13	56	219
Neurological Disease	-	12	59	147
Organismal Injury and Abnormalities	-	12	30	56
Cellular Growth and Proliferation	-	11	93	114
Connective Tissue Disorders	-	11	15	21
Cell Signaling	-	-	223	662
DNA Replication Recombination and Repair	-	-	183	194
Genetic Disorder	-	-	128	218
Nervous System Development and Function	-	-	98	166
Hematological Disease	-	-	92	89
Protein Synthesis	-	-	88	204
Dermatological Diseases and Conditions	-	-	82	175
Hematological System Development and Function	-	-	81	116
Lipid Metabolism	-	-	79	363
Cell-To-Cell Signaling and Interaction	-	-	76	122
Skeletal and Muscular Disorders	-	-	76	33
Immune Response	-	-	71	246
Cardiovascular Disease	-	-	70	80
Cellular Function and Maintenance	-	-	61	263
Embryonic Development	-	-	54	180
Endocrine System Development and Function	-	-	53	62
Immunological Disease	-	-	44	54
RNA Post-Transcriptional Modification	-	-	43	122
Nucleic Acid Metabolism	-	-	39	130
Reproductive System Development and Function	-	-	35	26
Tissue Development	-	-	34	73
Protein Trafficking	-	-	34	63
Protein Folding	-	-	32	31
Carbohydrate Metabolism	-	-	31	136
Cardiovascular System Development and Function	-	-	31	127
Organismal Development	-	-	29	30
Cellular Compromise	-	-	28	96
Organ Morphology	-	-	28	91
Vitamin and Mineral Metabolism	-	-	27	61
Skeletal and Muscular System Development and Function	-	-	25	33
Developmental Disorder	-	-	18	33
Inflammatory Disease	-	-	16	95
Nutritional Disease	-	-	16	30
Infectious Disease	-	-	14	65
Metabolic Disease	-	-	12	89
Viral Function	-	-	61	-
Reproductive System Disease	-	-	48	-
Ophthalmic Disease	-	-	32	-
Hair and Skin Development and Function	-	-	31	-
Organ Development	-	-	31	-
Cardiac Fibrosis	-	-	30	-
Viral Infection	-	-	30	-
Cardiac Enlargement	-	-	28	-
DNA Replication	-	-	20	-
Cardiac Pulmonary Embolism	-	-	17	-
Dermatological Diseases and Condition	-	-	17	-
Cardiac Necrosis/Cell Death	-	-	16	-
Digestive System Development and Function	-	-	16	-
Hepatic System Disease	-	-	14	-
Tumor Morphology	-	-	13	-
Protein Degradation	-	-	-	119
Energy Production	-	-	-	84
Gastrointestinal Disease	-	-	-	65
Behavior	-	-	-	61
Immune and Lymphatic System Development and Function	-	-	-	61
Renal Necrosis/Cell Death	-	-	-	33
Renal and Urological System Development and Function	-	-	-	32
Endocrine System Disorders	-	-	-	31
Cardiac Hypertrophy	-	-	-	30
Organismal Survival	-	-	-	28
Free Radical Scavenging	-	-	-	27
Respiratory System Development and Function	-	-	-	25
Tissue Morphology	-	-	-	23
Hepatic System Development and Function	-	-	-	21

Focus genes	12	98	1474	2887

*The same focus gene may exist in different top functions.

^§ ^The number of focus genes from the Qiagen_NRSP8 microarray is reported at each time point for each top function.

### PrV infection modifies the expression of genes involved in MHC antigenic presentation pathways

The expression of many genes belonging to the SLA class I antigenic presentation pathway was modulated during PrV infection according to the results of both microarrays (Table 5). SLA Ia genes were down-regulated from 4 h pi with the SLA/PrV microarray and from 8 h pi with the Qiagen-NRSP8 microarray. TAP1 and TAP2 genes, encoding molecules involved in peptide transport from the cytosol to the endoplasmic reticulum, were also down-regulated 8 h pi according to the results of the Qiagen-NRSP8 microarray. Surprisingly, TAP1 was up-regulated 8 h pi with the SLA/PrV microarray. PSMB8 (alias LMP7), one of the genes encoding immunoproteasome molecules was up-regulated from 4 h pi on the Qiagen-NRSP8 microarray.

**Table 5:** Subset of differentially expressed cellular genes at each time point.

**Pathway or function**	**Clone or oligo name**	**Gene symbol**	**Human refseq**	**Fold change**						**Microarray**
					
				**T0**	**T1**	**T2**	**T4**	**T8**	**T12**	
**Class I antigenic Pathway**	SS00013127	HLA-A	NM_002116	NS	-	-	-	-4.6	NS	Qiagen-NRSP8
	SS00001303	HLA-A	NM_002116	NS	-	-	-	-3.8	NS	Qiagen-NRSP8
	SCAN0007.O.20	SLA Ia	NM_002116	-	-	-	-4.9	-4.6	-4.5	SLA/PRV
	SCAN0032.G.07	SLA Ia	NM_002116	-	-	-	-	-4.0	-	SLA/PRV
	SCAN0010.J.21	SLA Ia	NM_002116	-	-	-	-	-	-5.9	SLA/PRV
	SCAA0099.O.04	SLA-7	NM_002116	-	-	-	-3.5	-3.6	-	SLA/PRV
	SS00000703	PSMB8	NM_148919	NS	-	-	5.5	7.8	NS	Qiagen-NRSP8
	SCAC0037.O.09	TAP1	NM_000593	-	-	-	-	3.9	-	SLA/PRV
	SS00010012	TAP1	NM_000593		-	-	-	-7.8	NS	Qiagen-NRSP8
	SS00002173	TAP2	NM_000544	NS	-	-	-6.9	-	NS	Qiagen-NRSP8
	SS00010176	MICB	NM_005931	NS	-	-	11.1	11.3	NS	Qiagen-NRSP8

**Class II antigenic pathway**	SS00000697	HLA-DMB	NM_002118	NS	-	-	-	-7.7	NS	Qiagen-NRSP8
	SS00000661	HLA-DOA	NM_002119	NS	-	-	-	5.2	NS	Qiagen-NRSP8
	SS00000973	HLA-DQA1	NM_002122	NS	-	-	-	5.2	NS	Qiagen-NRSP8
	SCAB0137.B.15	HLA-DOB	NM_002120	-	-	-	-4.0	-	-	SLA/PRV
	SCAC0044.H.08	HLA-DMB	NM_002118	-	-	-	-4.6	-	-	SLA/PRV
	SS00000824	CIITA	NM_000246	NS	-	-	-	3.3	NS	Qiagen-NRSP8

**Immunity (other genes)**	SS00010182	CD4	NM_000616	NS	-	5.1	14.6	14.1	NS	Qiagen-NRSP8
	SS00009986	CD69	NM_001781	NS	-	-	-3.1	-5.9	NS	Qiagen-NRSP8
	SS00006217	CLEC2L	XM_498242	NS	-	3.2	11.8	16.1	NS	Qiagen-NRSP8
	SS00009653	CLEC5A	NM_013252	NS	-	-	-4.0	-6.9	NS	Qiagen-NRSP8
	SS00002913	IK	NM_006083	NS	-	-	-	5.0	NS	Qiagen-NRSP8
	SS00010183	ICAM1	NM_000201	NS	-	-	-4.2	-9.2	NS	Qiagen-NRSP8
	SS00003797	IFNAR2	NM_207585	NS	-	-	3.5	7.9	NS	Qiagen-NRSP8
	SS00010797	IFNGR2	NM_005534	NS	-	-	-	-6.1	NS	Qiagen-NRSP8
	SS00002396	IRF1	NM_002198	NS	-	-	-5.0	-9.3	NS	Qiagen-NRSP8
	SS00009562	IRF2	NM_002199	NS	-	-	-4.4	-10.8	NS	Qiagen-NRSP8
	SS00000183	IRF3	NM_001571	NS	-	-	-	-3.9	NS	Qiagen-NRSP8
	SS00003318	IRF5	NM_032643	NS	-	-	-3.5	-7.9	NS	Qiagen-NRSP8
	SS00000904	IFNA6	NM_021002	NS	-	-	-	-7.2	NS	Qiagen-NRSP8
	SS00010817	IFI6	NM_002038	NS	-	-	6.5	12.5	NS	Qiagen-NRSP8
	SS00010811	IFI30	NM_006332	NS	-	-	-	-6.1	NS	Qiagen-NRSP8
	SS00001608	ISGF3G	NM_006084	NS	-	-	-	3.9	NS	Qiagen-NRSP8
	SS00009843	IL12A	NM_000882	NS	-	-	-3.3	-17.1	NS	Qiagen-NRSP8
	SS00009841	IL12B	NM_002187	NS	-	-	-7.9	-17.1	NS	Qiagen-NRSP8
	SS00008021	TLR8	NM_016610	NS	-	-	-4.6	-5.9	NS	Qiagen-NRSP8
	SCAA0015.B.22	PPIA	NM_021130.3	-	-	-	-3.9	-4.8	-4.8	SLA/PRV
	SCAU0001.B.05	PPIA	NM_021130.3	-	-	-	-3.6	-4.3	-4.7	SLA/PRV
	SS00003892	PPIL1	NM_016059	NS	-	-	-	3.2	NS	Qiagen-NRSP8
	SS00001759	PPIL2	NM_148176	NS	-	-	-	4.1	NS	Qiagen-NRSP8
	SS00001178	PPIF	NM_005729	NS	-	-	-	-3.8	NS	Qiagen-NRSP8
	SS00001241	PPIG	NM_004792	NS	-	-	-	-4.3	NS	Qiagen-NRSP8
	SS00005782	PPIH	NM_006347	NS	-	-	-	5.1	NS	Qiagen-NRSP8
	SS00006083	FKBP3	NM_002013	NS	-	-	-	-8.3	NS	Qiagen-NRSP8
	SS00002702	FKBP4	NM_002014	NS	-	3.6	13.0	14.3	NS	Qiagen-NRSP8

**Apoptosis**	SS00003686	BNIP1	NM_001205	NS	-	-	-	-6.1	NS	Qiagen-NRSP8
	SS00000546	BAK1	NM_001188	NS	-	-	-	-4.3	NS	Qiagen-NRSP8
	SS00010935	BCLAF1	NM_014739	NS	-	-4.0	-4.6	-6.6	NS	Qiagen-NRSP8
	SS00001150	BCL2L1	NM_138578	NS	-	-	-3.4	-6.5	NS	Qiagen-NRSP8
	SS00005026	BCL2L14	NM_138723	NS	-	-	-	-5.8	NS	Qiagen-NRSP8
	SS00000872	CASP1	NM_033293	NS	-	-	3.6	5.0	NS	Qiagen-NRSP8
	SS00000520	CASP3	NM_032991	NS	-	-	-	-0.7	NS	Qiagen-NRSP8
	SS00004783	CASP7	NM_001227	NS	-	-	-	6.7	NS	Qiagen-NRSP8
	SS00003624	CARD6	NM_032587	NS	-	-	3.5	6.6	NS	Qiagen-NRSP8
	SS00000561	DDX58	NM_014314	NS	-	-3.1	-4.3	-8.3	NS	Qiagen-NRSP8
	SS00000329	FAF1	NM_007051	NS	-	-	-	5.8	NS	Qiagen-NRSP8
	SS00012494	FAIM2	NM_012306	NS	-	-	-4.6	-10.7	NS	Qiagen-NRSP8

ER* stress	SS00000384	EIF2A	NM_032025	NS	-	-	3.7	7.1	NS	Qiagen-NRSP8
**Pathway**	SS00008645	XBP1	NM_005080	NS	-	-	-	-6.4	NS	Qiagen-NRSP8
	SS00004223	ATF4	NM_182810	NS	-	-	-	-4.1	NS	Qiagen-NRSP8
	SS00001308	HSPA8	NM_006597	NS	-	-	-	3.6	NS	Qiagen-NRSP8

**Nucleic acid**	SCAB0073.K.18	HIST1H2BK	NM_080593	-	-	-	-3.9	-6.8	-4.4	SLA/PRV
**Binding**	SCAA0122.E.09	HIST1H2AL	NM_003511	-	-	-	-3.9	-6.0	-3.7	SLA/PRV
	SCAB0057.M.21	HIST1H4J	NM_021968	-	-	-	-5.1	-4.4	-4.0	SLA/PRV
	SCAB0015.G.08	HIST3H2BB	NM_021968	-	-	-	-	-4.2	-5.4	SLA/PRV
	SS00005067	HDAC2	NM_001527	NS	-	4.6	8.3	9.1	NS	Qiagen-NRSP8
	SS00004784	HDAC3	NM_003883	NS	-	-	-5.5	-10.9	NS	Qiagen-NRSP8
	SS00007434	HDAC6	NM_006044	NS	-	-	-3.7	-8.4	NS	Qiagen-NRSP8
	SS00003586	HDAC9	NM_014707	NS	-	-3.5	-4.8	-10.6	NS	Qiagen-NRSP8
	SS00004682	HDAC10	NM_032019	NS	-	-	-	3.9	NS	Qiagen-NRSP8
	SS00007084	STAT1	NM_007315	NS	-	-	-	7.6	NS	Qiagen-NRSP8
	SS00008286	STAT3	NM_139276	NS	-	-	-	-4.4	NS	Qiagen-NRSP8
	SS00008435	STAT5B	NM_012448	NS	-	-	-5.3	-9.9	NS	Qiagen-NRSP8
	SS00004427	STAT6	NM_003153	NS	-	-	-	-4.6	NS	Qiagen-NRSP8

**Cytoskeleton**	SS00003440	ACTC1	NM_005159	NS	-	-	-	-8.9	NS	Qiagen-NRSP8
	SS00013114	ACTG1	NM_001614	NS	3.3	6.9	19.4	18.2	NS	Qiagen-NRSP8
	SS00002774	ACTL6A	NM_004301	NS	-	-	-	3.6	NS	Qiagen-NRSP8
	SS00005476	ACTRT2	NM_080431	NS	-	-	-	-7.2	NS	Qiagen-NRSP8
	SS00007431	ACTA4	NM_00492	NS	-	-	-3.7	-3.9	NS	Qiagen-NRSP8
	SS00011046	MYO1D	NM_015194	NS	-	-	-4.7	-10.2	NS	Qiagen-NRSP8
	SS00007818	MYO5A	NM_000259	NS	-	-	-3.7	-9.8	NS	Qiagen-NRSP8

* ER: Endoplasmic Reticulum

NS: Not Studied

-: not differentially express

Unexpectedly, our results show that transcript levels of genes belonging to the MHC class II antigenic presentation pathway were also modulated during PrV infection. Expression of SLA-DOB and SLA-DMB decreased at 4 h pi according to the results from the SLA/PrV microarray (Table 5). SLA-DMB was also down-regulated with the Qiagen-NRSP8 microarray 8 h pi while SLA-DOA, SLA-DQA1 and CIITA were up-regulated at this time point (Table 5).

### Immune response, apoptosis, nucleic acid binding and actin cytoskeleton pathways are modulated during PrV infection

Among all the biological processes (Table 3) and top functions (Table 4), shown to be regulated during PrV infection, we examined in greater detail genes differentially expressed in four pathways i.e. immune response, apoptosis, nucleic acid binding and actin cytoskeleton (Table 5).

For genes involved in immune response, we observed that CD4 and CD69 were up- and down-regulated from 4 h pi, respectively (Table 5) and that several chemokine ligand and interleukin genes such as IL12A, IL12B and IL17 were down-regulated at 4 and 8 h pi. These observations have a poor biological significance. Since the relevant gene products are known to be specific of immune cells, it is probable that these transcript expressions are not correlated with significant protein synthesis in the present epithelial cell context. Among the genes involved in interferon-mediated immunity, many were modulated during PrV infection (Table 5) i.e. IFNAR2 and IFI6 transcript levels increased from 4 h pi and ISGF3G transcript levels at 8 h pi. The expression of IRF1, IRF2and IRF5 appeared down-regulated from 4 h pi and that of IRF3 at 8 h pi. IFNA6, IFI30 were down-regulated 8 h pi while IFNG, which was included in the SLA/PrV probe set, was not detected as a differentially expressed gene. In addition, the expression of TLR8, involved in recognition of viral nucleic acid binding was decreased at 8 h pi. Immunophilin genes were also regulated during infection. From 4 h pi PPIA (alias Cyclophilin A) was down-regulated (Table 5) and at 8 h pi PPIF and PPIG were down-regulated while PPIH was up-regulated.

For the apoptosis pathway, genes belonging to the BCL-2 molecule family, FAIM2, CASP1 and CASP3 were down-regulated whereas CASP7 and NF-KB2 were up-regulated. Transcript levels of JAK1, XBP1, ATF4 and HSPA5 were decreased at 8 h pi. HSPA1A, HSPA1B, HSPA2, HSPA4, HSPA4K and HSPA8 were up-regulated at 8 h pi and EIF2A from 4 h pi. HSPA6 was first down-regulated at 2 h pi and then up-regulated from 4 h pi. Several differentially expressed genes, which belong to the apoptosis pathway, were also involved in the stress response.

Among the differentially expressed genes that play a role in nucleoside, nucleotide and nucleic acid binding, the expression of histone genes HIST1H2AL, HIST1H4J, HIST1H2BK was decreased from 4 h pi. Expression of several histone deacetylases was also regulated during infection: HDAC2 and HDAC10 expression levels increased, while HDAC3, HDAC6, and HDAC9 expression decreased. HDAC2 and HDAC9 were regulated early from 2 h pi. Several genes encoding signal transducers and activators of transcription (STAT1, STAT3, STAT5B and STAT6) were down-regulated during PrV infection.

Within the actin cytoskeleton pathway, ACTG1 was up-regulated very early i.e. as soon as 1 h pi. Other genes such as ACTC1, ACTRT2, ACTA4, MYO1D and MYO5A were all down-regulated from 4 or 8 h pi and ACTL6A was up-regulated at 8 h pi.

### Validation of microarray results by quantitative real-time PCR (qRT-PCR)

Five genes involved in the presentation antigen class I pathway were studied by qRT-PCR: SLA Ia, TAP1, TAP2, PSMB8 and PSMB9. PPIA, down-regulated during infection, and TNF, even if not detected as differentially expressed in our transcriptome experiment, were also chosen for validation (Table 6). qRT-PCR were performed for a subset of conditions at 0, 2, 4, 8, and 12 h pi (see materials and methods). We confirmed that SLA Ia genes were down-regulated during infection from 8 h pi. We also observed a clear down-regulation of TAP1 and TAP2 from 8 and 4 h pi, respectively. An early down-regulation of PSMB8 and PSMB9 was detected before 2 h pi. TNF was strongly up-regulated from 4 h pi and PPIA was down-regulated from 2 h pi.

**Table 6:** Cellular gene expression study by qRT-PCR.

	**Change (n-fold)**					
	
**Gene**	MI*_T12/MI*_T0	I**_T0/MI*_T0	I*_T2/MI**_T0	I**_T4/MI*_T0	I**_T8/MI*_T0	I**_T12/MI*_T0
**TNF**	1.6	2.3	2.6	14.7	14.5	10.5
**PPIA**	1.2	1.1	0.7	0.2	0.1	0.1
**SLA Ia**	0.7	0.9	-	-	0.6	0.5
**TAP1**	-	-	-	-	0.8	0.6
**TAP2**	1.2	1.2	1.0	0.8	0.5	0.4
**PSMB9**	0.8	0.6	0.6	0.7	0.3	0.3
**PSMB8**	-	0.6	0.7	0.6	0.3	0.2

- - no detected difference between two samples

- *: mock-infected

- **: PrV infected

### Cell surface expression of MHC class I and MHC class II molecules on PK15 cells during PrV infection

**Figure 5:**
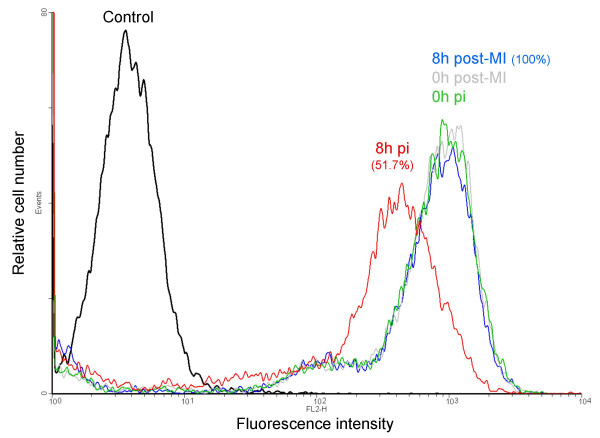
**SLA I cell surface decrease on PrV infected PK15 cells. **Histogram overlays of MHC class I expression detected by the mAb PT85A are shown. The number in red represents the percent mean fluorescence intensity [(mean channel fluorescence of the infected sample at 8 h pi/mean channel fluorescence of the mock-infected cells at 8 h pi) × 100]. The results represent one of three representative experiments.

Since our experiments, as well as other studies [6,7], have clearly indicated a down-regulation of the MHC class I genes during PrV infection, we checked, by flow cytometry, for a down-regulation of surface MHC class I molecules on PrV infected PK15 cells at 8 h pi. To visualize infected cells, we used, in the same experimental conditions, a recombinant PrV strain (derived from NIA3) expressing the green fluorescent protein (GFP). Ninety percent of the cells appeared infected and 73% of these infected cells expressed surface MHC class I molecules on their surface while 89.1% and 83.9% of the mock-infected cells expressed MHC class I molecules at 0 and 8 h pi, respectively (data not shown). The MHC class I mean fluorescence intensity of infected cells at 8 h pi was 50.9% of that of mock-infected cells (mean of three experiments) thus confirming a clear decrease of MHC class I molecules expression on the surface of infected cells (Figure 5). As a control, we observed that the expression of tubulin, detected by Western blot, remains unchanged even 8 h pi in PK15 cells (data not shown).

Since a significant variation in MHC class II transcript levels during infection was detected in our transcriptome analysis, we also analyzed the expression of MHC class II molecules on the surface of PK15 cells. Our results show that 5.5% of mock-infected cells (at 0 and 8 h pi) and infected cells (0 h pi) expressed surface MHC class II molecules. However, we could not detect any differential expression between infected and mock-infected cells at 8 h pi.

## Discussion

### A joint PrV-porcine epithelial cell transcriptomic approach

This work is the first study of PrV transcriptome expression during the time course of infection. Moreover, it is the first time that the gene expressions of both PrV (NIA3 strain) and porcine cells during infection are analyzed simultaneously and we demonstrate that virus and host cell transcriptome modifications can be examined with a unique microarray combining viral and host cell probe sets. Indeed, a majority of transcriptomic studies have focused either on host or on pathogen gene expression profiling [11,22,23] and only a few studies report the simultaneous detection of pathogen and mammalian host transcriptomes i.e. *Plasmodium berghei *ANKA and mouse [24] and EBV-NK/T cell lymphoma and man [25]. Our work confirms the feasibility and the relevance of this kind of approach to establish a direct link between pathogen and cellular gene expression. In order to explore porcine cellular gene expression with even more detail, we chose to supplement the SLA/PrV microarray with the Qiagen-NRSP8 microarray. The sensitivity of each microarray differed according to the nature of the probes (70-mer oligonucleotides versus DNA/cDNA) as shown by comparative studies [26]. Seventy-mer oligonucleotides give better results in terms of specificity and sensitivity compared to cDNA microarrays and this could explain some discrepancies observed between both microarrays in particular for the TAP1 gene [27] as confirmed in our study. With this integrated approach, a parallel increase in the number of differentially expressed PrV and cellular genes was detected illustrating the viral and cellular transcript modifications during infection.

### A picture of PrV gene transcription during PK15 cell line infection

In our experimental conditions, we obtain a picture of the global PrV gene transcription during the lytic cycle. PrV transcription was monitored in single cycle conditions using a high MOI that guarantees that more that 90% cells are infected. Despite the presence of nested transcription units in PrV preventing the design of probes specific of unique transcripts for some genes, we were, however, able to confidently report viral gene expression for probes specific of unique viral transcripts and draw a general picture of PrV transcription during the time course of infection. As expected, the expression of most viral genes increased during infection. Our results show that a notable increase in transcript levels and in the number of differentially expressed viral probes, detected from 4 h pi, correlates with viral growth and thus coincides with the beginning of the release of extracellular progeny (see Figure 2, Figure 3, Table 1 and Table 2). It has already been reported that the beginning of viral progeny usually occurs between 4 and 5 h pi but without any description of the global viral transcription [1]. We observe a continuous increase in transcript levels and in the number of differentially expressed viral probes between 4 and 8 h pi followed by a stabilization when virion production is maximum. This suggests that the transcriptional machinery is fully active at 8 h pi thus permitting a massive virion production. All the different classes of viral transcripts are represented from 4 h pi (non structural, capsid, tegument and envelope protein transcripts). The molecular hallmark of herpesvirus infection is a temporally ordered gene transcription. As for other herpesviruses, the PrV genes are subdivided in three main classes of successively expressed transcripts: immediate early, early and late transcripts [1]. After binding of the viral particle and fusion of the virion envelope with the cell membrane, the release of capsid and tegument proteins into the cell and the takeover of host cell protein synthesis machinery, the IE180 protein encoded by the immediate early gene is expressed in the cytoplasm and translocated to the nucleus. This protein can further transactivate the RNA polymerase II mediated transcription of early genes including transactivators of transcription (EP0, US1, UL54) and proteins essential for viral replication (UL5, UL8, UL9, UL29, UL30, UL42, UL52, UL28, UL39, UL40, UL23, UL50, UL12, UL2) [1]. The expression of IE180 has been reported to begin between 40 min and 1 h pi and last until 3 h pi [1]. In our experiment, the IE180 probe (IEP2) was differentially expressed only at 4 h pi, when the transcript level probably reaches its peak value. This suggests that a low level of IE180 transcripts is sufficient to induce the transcription of early genes. In this experiment, the differential expression of US1 and UL29 was detected as early as 1 h pi but other early genes appeared differentially expressed later. Interestingly, the UL49.5 probe corresponding to the gN protein, responsible for TAP inhibition, was differentially expressed at 1 h pi, even if this gene is not described as an early gene. The synthesis of late proteins, such as capsid (UL6, UL18, UL19, UL25, UL35 and UL38), tegument (UL11, UL13, UL16, UL21, UL36, UL37, UL37, UL41, UL46, UL47, UL48, UL49, UL51, US3, US2) and envelope proteins (gE, gI, gD, gM, gH, gC, gB, gN, gK) are reported to occur during the PrV replication cycle [1]. In our study, the two late transcripts UL6 and UL22 encoding the gH protein were differentially expressed as early as 2 h pi. The four latest differentially expressed genes mostly encoded envelope or tegument proteins, except UL9.

We described for the first time a global analysis of PrV gene transcription using a microarray. The results of our analysis is consistent with what is known about PrV viral cycle and with the kinetic classification of individual transcripts [1]. Similar approaches have been developed for other alphaherpesviruses such as HSV-1 [28,29] and Varicella-Zoster virus (VZV) [28,29]. It is difficult to compare our results with those obtained in the VZV study because this viral system does not allow cell infection under single cycle synchronized conditions (one limitation of this viral system), which is required to establish reliable kinetics of viral gene expression. However our results are consistent with the transcriptomic study reported for HSV-1 [28,29]. It is clear from figure 3 that PrV early homologues of HSV-1 immediate early genes (EP0, UL54, US1) and early genes (UL23 encoding thymidine kinase, UL30 encoding a DNA polymerase subunit, UL39 encoding the large subunit of ribonucleotide reductase) are expressed at early times before most of the late genes encoding structural proteins. A clear distinction between immediate early, early and late genes for PrV will require transcriptomic analysis in the presence of the translation inhibitor cycloheximide (to identify immediate early genes) or the viral DNA replication inhibitor phosphonoacetic acid (to distinguish early and late genes) as was done for HSV-1 [28,29].

### PrV and cellular shutoff

A cellular shutoff during infection has been described for herpesviruses including PrV [1]. In our experiment, a shutoff of PK15 genes is observed during infection since many cellular genes are down-regulated between 4 and 12 h pi. In contrast, at the 4 h time point, 42.5 % of the viral genes are up-regulated. Our transcriptomic analyses reveal that the shutoff occurs in porcine cells earlier than that previously reported in other transcriptome studies i.e. between 8 and 12 h pi in rat embryonic fibroblasts and from 6 h pi in human embryonic kidney cells [12,14]. It is assumed that the virion host shutoff protein (vhs/UL41 transcript) causes cellular shutoff. The vhs protein is an RNAse located in the viral tegument, which degrades host and viral RNA just after infection for HSV-1 [30]. Unlike HSV-1, it has been suggested that for cellular shutoff, PrV requires a fresh round of viral protein synthesis explaining the observed delayed shutoff [9]. In our experiment, UL41 transcripts appear to be differentially expressed only at 8 h pi suggesting that the vhs activity can be attributed to the newly synthesized proteins and not to the vhs proteins present in the virion tegument at the moment of infection and that the vhs protein should be active at low level. The activity of HSV-1 vhs is modulated by the UL48 product (VP16), which can bind to vhs to allow viral mRNA accumulation [31]. However, our study does not show any differential expression of the UL48 transcript.

### PrV infection and immune evasion strategies

To evade host response PrV develops several strategies that probably disturb different biological pathways including the MHC class I presentation pathway. We observed a decrease of SLA-Ia and TAP2 transcript levels in PK15 cells infected with the PrV NIA3 strain as previously reported in infected PK15 and bovine kidney cells respectively (IND-F PrV strain) [9]. A down-regulation of TAP1 and TAP2 genes encoding immunoproteasome catalytic subunits, PSMB8 and PSMB9, involved in the MHC class I antigenic presentation pathway was also detected in our experiment. Moreover, we checked that at 8 h pi the PK15 cells expressed 50% less MHC class I proteins than mock-infected cells in our culture conditions. These results confirm previous reports describing the reduced capacity of infected cells to present viral peptides to CTL [6,7]. The viral gene UL49.5, encoding the gN protein, is one of the earliest differentially expressed genes in our study (from 1 h pi). This viral protein has been shown to inhibit TAP activity and induce degradation of TAP molecules by the proteasome [5,8,32]. Our results strongly suggest a very early production of gN protein and agree with the detection of TAP inhibition from 2 h pi [7]. This TAP inhibition has been shown to be independent of vhs activity [9] and we demonstrate here that UL41 encoding vhs is differentially expressed later than UL49.5, indicating two successive steps i.e. TAP inhibition followed by cellular shutoff. Since the level of several transcripts involved in the MHC class I presentation pathway (MHC class Ia, TAP1, TAP2, PSMB8 and PSMB9) decreased, it is possible that PrV has developed complementary strategies to evade this pathway i.e. turning off the peptide pump with inhibition of TAP activity and transcription alteration of key players [5]. Other viruses, such as the human cytomegalovirus, down-regulate the transcription of key players of the MHC class I antigen presentation pathway [5]. Unexpectedly, some MHC class II genes were also regulated during PrV infection in PK15 cells. In particular, a down-regulation of class II-like chaperones SLA-DOB and SLA-DMB was observed. MHC class II molecules that are constitutively expressed on professional antigen presenting cells (APCs), present peptides derived from exogenous antigens to CD4+ T-helper cells playing an important role in the induction and maintenance of CTL immunity. Epithelial cells can also constitutively express MHC II molecules but at a lower level than professional APCs [33]. In our experiment, we detected a small subpopulation of uninfected PK15 cells constitutively expressing MHC class II molecules. However, we did not detect modifications of the MHC class II expression at the cell surface. Since a down-regulation of constitutive and IFNγ induced HLA class II expression has been observed in cells infected by other herpesviruses [33], our preliminary data suggest that it would be highly relevant to explore how PrV may interfere with the MHC class II presentation pathway in professional APCs.

In addition to genes belonging to MHC antigen presentation pathways, several other genes, playing a role in anti-viral response are regulated during PrV infection such as genes belonging to the IFN signaling pathway. Indeed, it has been reported that in primary rat fibroblasts, PrV infection could suppress the establishment of the IFNβ-induced viral state [13]. IRF3, which is a transcriptional factor involved in IFNβ production by epithelial cells, is down-regulated together with a set of other IRF. Constitutively expressed in the cytosol, IRF3 is phosphorylated during herpesvirus infection and translocated into the nucleus to target the IFNβ promoter. Many viruses interfere with IRF activities [34]. A decrease of IRF1 mRNA and protein levels has also been detected in cells infected with hepatitis C virus, resulting in the transcriptional repression of several IFN-stimulated genes [35]. In addition, TNF-alpha, which is a multifunctional cytokine with potent antiviral activities and which mediates protection against HSV-1 in the mouse [36] was analyzed by qRT-PCR. A strong up-regulation of TNF was detected from the beginning of PrV infection and until 12 h pi. These results suggest that the TNF transcription increase that is usually expected during an infection is not suppressed by PrV infection and that the cellular shutoff does not target TNF.

### Other cellular pathways modulated during PrV infection

Our transcriptome analysis confirms that many other biological processes and functions are modulated during PrV infection in porcine PK15 cells as previously observed in rat embryonic fibroblast and human embryonic kidney cells [12,14]. We have focused our study on a limited number of pathways and genes. Interestingly, we observe both by transcriptome analysis and qRT-PCR that PPIA gene expression is clearly down-regulated during PrV infection. PPIA encodes cyclophilin A, a peptidyl-prolyl isomerase, which catalyzes the isomerization of peptide bonds from the *trans *to *cis *form at proline residues and facilitates protein folding [37] and which acts as a cytosolic molecular chaperone. Cyclophilins have been discovered because of their high affinity for cyclosporine, an immuno-suppressive drug, which prevents allograft rejection. This immunosuppressive effect is due to the calcineurin inhibition by a cyclosporin-cyclophilin complex. Calcineurin is required for transcriptional activation of many cytokines in stimulated T cells. Cyclophilin A can also interact with HIV-1 Gag polyprotein and is involved in HIV-1 replication kinetics and modifies the infectivity of HIV-1 virions in Jurkat T cells [38]. Indeed, virions produced by PPIA^-/- ^cells are less infectious than virions produced by PPIA^+/+ ^cells. Since we observed a down-regulation of PPIA before the global cellular shutoff, cyclophilin A may be a target for PrV and play a role in infection via an unknown mechanism.

Several cellular genes involved in apoptosis were regulated during PrV infection such as BCl-2 molecules and caspases. Viral infection of mammalian cells tends to generate proapoptotic signals to limit viral replication but viruses and, in particular, herpesviruses produce molecules acting as modulators of apoptosis [1]. Thus, US3 products from PrV play an anti-apoptotic role [39]. The PIKNIA3 probe specific to US3 long and short isoform transcripts was up-regulated from 8 h pi in our experiment, suggesting a possible late antiapoptotic role of the US3 products. In addition, PrV genes homologous to other HSV-1 antiapoptotic genes may also possess an antiapoptotic role such as UL54 or US1 [1]. UL54 was not differentially expressed in our experiment in contrast to US1, which was up-regulated very early as soon as 1 h pi.

Genes belonging to nucleic acid metabolism were differentially expressed from very early time points. A repression of many genes encoding histones and nucleosome components occurred in PK15 cells during infection. These results are concordant with a former study, which has shown a gradual inhibition of histone synthesis in RK13 rabbit cells during PrV infection [40]. We also observed a modulation of many histone deacetylases (HDAC). Acetylation of newly synthesized histones is required for their assembly into nucleosomes by histone chaperones and regulates the formation of heterochromatin that is critical for cellular gene transcription. US3, which was up-regulated from 8 h pi in our experiment, can suppress histone acetylation during HSV1 infection [41]. Indeed, PrV US3 could also inhibit histone acetylation during infection.

PrV infection regulates the expression of several genes involved in actin cytoskeleton signaling. A probe specific to ACTG1 was strongly up-regulated as soon as 1 h pi and reached a peak at 4 h pi. Cytoskeleton actin is involved in PrV assembly and in virus movement within the host cell. In particular, viral capsids can travel along nuclear actin filaments using myosin-directed transport in neurons but also in PK15 cells [42]. Moreover, actins present in the nucleus participate in transcription [43]. Among PrV proteins, the US3 protein kinase contributes to cellular cytoskeleton modifications via the formation of actin- and microtubule-containing cell projections, a phenomenon associated with an increase of PrV intercellular spread [44].

## Conclusion

The originality of our approach lies in the simultaneous investigation of transcript levels of both host and pathogen genomes using a partial generic microarray and a dedicated microarray (SLA/PrV) combining all the PrV genes and probes from the SLA complex. It is now necessary to extend our analysis of the interactions between PrV and porcine cells to other target cells, such as immature dendritic cells (iDC) that are the first immune cells interacting with the virus. This kind of approach should also be efficient (i) to study viral and cellular gene expression using mutant viruses in order to better understand the role of each viral gene and (ii) to help identify species or strain specific transcriptomic signatures in host cells.

## Methods

### Cells, viruses and infection

The PK15 cells used in this study, for both viral stock production and virus-cell interaction experiments, were propagated in the H-MSM aproteic synthetic medium (R. L'Haridon, unpublished results) without serum. This medium consists in Eagle's Minimum Essential Medium (EMEM) supplemented with appropriate amounts of amino-acids, sugars, vitamins, salts and organic acids and without any additional hormone, natural or recombinant protein or growth factors. The PK15 cells underwent at least 40 passages in these conditions prior to this study.

The virulent wild type NIA3 strain of Pseudorabies virus as well as its GFP expressing derivative were grown by infecting confluent PK15 cell monolayers in 175 cm^2 ^flasks (Corning, France) at a MOI of 0.1. After a 48 h growth period, the cell culture medium, containing progeny virions, was collected, chilled on ice and clarified by centrifugation at 4°C. Virions were purified by ion exchange chromatography on Sartobind S cation exchanger membranes (Sartorius, Palaiseau, France) as described previously [45] except that SingleSep minicapsules were used instead of the MA100 device. After concentration by ultracentrifugation (Beckman SW41 rotor, 25,000 rev per min, 1 h, 4°C) purified virions were resuspended in TBSal buffer (200 mM NaCl, 2.6 mM KCl, 10 mM Tris-HCl, pH 7.5, 20 mM MgCl2, 1.8 mM CaCl2) and stored in aliquots at -80°C.

Infectious virus titers in purified stocks or cell culture supernatants were determined by plaque assay on PK15 cells grown in standard conditions (EMEM containing 10 % fetal calf serum) as described previously [46].

For virus-cell interaction experiments, aliquots of PK15 cells were seeded in 50 mm Petri dishes. We used the same batch of cells to prepare all the aliquots of the same time-course replicate experiment. When cells reached confluence, growth medium was removed and replaced by the inoculum (purified virions at a MOI of 20 diluted in a small volume of fresh medium) for infection by the mock-inoculum (virus resuspension buffer diluted the same way) for mock-infection. After a 45 min adsorption period at room temperature, inoculums and mock-inoculums were removed and replaced, after a single rinse, by H-MSM. At this time (considered as T0) monolayer cultures were further incubated at 37°C for the time required before RNA extraction. For flow cytometry experiments (see below), we used the same procedure except that the cells were mock-infected or infected with a recombinant PrV strain (derived from the NIA3 strain) that constitutively expresses the GFP under the control of the immediate-early promoter of HCMV (M. Cochet and F. Lefèvre, unpublished data). Cells were analyzed 8 h post-infection and we performed three replicate experiments.

### RNA isolation

Cells were harvested for RNA extraction just after infection or mock-infection (time 0), and 1, 2, 4, 8 and 12 hours after infection or mock-infection. RNA was extracted with the Trizol-chloroform method (Invitrogen, France), dissolved in 40μl DEPC treated water and quantified (Nanodrop, nyxor Biotech). For quantitative real-time RT-PCR, RNA was treated with DNase I and cleaned to remove any DNA contamination (DNase set and cleanup, Qiagen, France). RNA quality was checked on the Bioanalyzer Agilent (Agilent Technologies, France) and RNA with a RIN score between 8 and 10 were labeled and used in microarray or qRT-PCR experiments. The RNA were diluted at 1μg/μl final concentration and stored at -80°C.

### Design and production of the SLA/PrV microarray

The SLA/PrV microarray, composed of cDNA and sub-cloned exons, is a dedicated array containing porcine and PrV probes, which was produced by the CRB-GADIE (Centre de Ressources Biologiques pour la Génomique des Animaux Domestiques et d'Intérêt Economique, LREG, INRA, Jouy-en Josas, France). The list of genes present in the human SLA orthologous region (6p21-p23) established using the human sequence draft (May 2004) on UCSC web site [47] and porcine cDNA clones were selected when they were available in the AGENAE library [19] or in two American libraries MARC1PIG and MARC2PIG constructed by the United States Department of Agriculture (USDA). When no porcine cDNA clone was found, exons were identified by comparison between ESTs and genomic DNA using the ICCARE comparison mapping tool [48,49]. Primers were designed with Primer 3 [50].

To design the PrV amplicons, we first established a complete composite sequence of the PrV genome by merging genomic sequences available in Genbank from seven strains of PrV. Positions of the 5' and 3' ends of viral transcripts (true or putative) as well as ORFs were established according to published data or Genbank annotations (F. Lefèvre, unpublished). The location of the 80 amplicons, at least one per transcript, was chosen according to this map, generally close to the 3' end of the transcripts (Figure 1). In the case of nested transcription units, we designed as many amplicons as the number of nested transcripts so that each amplicon was located between the 5' ends of two consecutive transcripts. We used Primer 3 to design primers for amplification (30 cycles: 30 sec at 94°C, 30 sec at 60°C and 30 sec at 72°C, in the presence of 10% DMSO). PrV probes targeting 70 viral genes [see Additional file 3] and pig exons were subcloned in PGEM-T easy plasmid (Promega, France) and individual clones checked for correct amplification by sequencing. For the SLA and immune clones spotted on the SLA/PrV slides, sequence homology was checked by multi-alignment (BLAST) against human and pig EST databases [21,51]. The SLA/PrV microarray probes were reannotated taking into account the most significant BLAST results and its final geneID file was used in the analysis.

Plasmid clones bearing cDNAs, sub-cloned exons or PrV amplicons were used as templates for preparative PCR (Taq PCR Master Mix Kit, Qiagen, France) with universal primers located in the vector (35 cycles, same conditions as above for PrV amplicons and 30 sec at 94°C, 30 sec at 60°C and 2 min at 70°C for other probes). After purification (Multiscreen-PCR plates, Millipore, France), PCR products were checked on 1% agarose gel, quantified using Fluoroskan Ascent (Thermo Fischer Scientific, France), evaporated and pellets were resuspended in 13 μl SSC3X. Forty-six control spots were added to the slide (Lucidea Universal Scorecard, Amersham Biosciences, France). Two identical arrays were spotted on slides (25 × 75 mm, UltraGAPS Coated Slides, Corning) with a 16 needle spotter (Microarraywriter Pro, Virtek, France). After spotting, slides were first treated with steam to homogenize the hydration of spots. Then, spotted DNA was denatured (5 sec at 100°C) and UV fixed (300 mJ). Slides were stored in dry atmosphere before use.

The SLA/PrV DNA/cDNA microarray platform has been submitted to the Gene Expression Omnibus (GEO) repository [52]. The accession number is GPL5622.

### The generic porcine array

The second microarray is a commercial generic microarray spotted on slides (Qiagen-NRSP8) and contains 13297 oligonucleotides (70-mers) specific of 8541 porcine genes [17]. The microarrays used in this study were provided by Dr Max Rothschild (Department of Animal Science, Iowa State University). We used the annotation of Qiagen-NRSP8 slides given by Zhao and collaborators [17].

### RNA labeling, hybridization scheme, microarray scanning and signal quantification

Five μg of each RNA were reverse-transcribed and labeled with Cy3 and Cy5 (reagents: Pronto kit, Corning and Amersham, Biosciences). Labeled targets were quantified (Nanodrop, Nyxor Biotech, France), evaporated and the pellets were resuspended in hybridization buffer (Pronto Kit, Corning, France) at a final concentration of 2 pmoles/μl. Control RNA (Spikes, Lucidea Universal Scorecard, Amersham Biosciences, France) specific to the control spots on the SLA slides were labeled together with total RNA.

**Figure 6:**
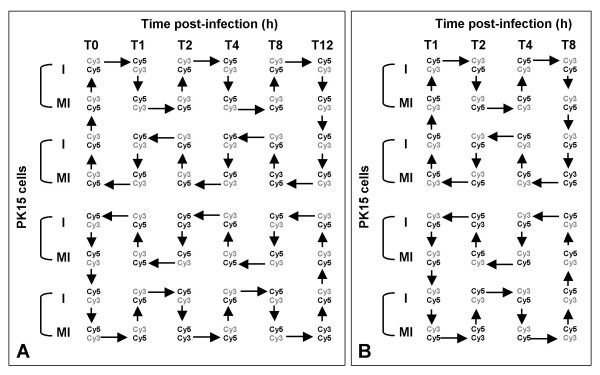
**Hybridization scheme of the SLA/PrV and the Qiagen-NRSP8 microarrays**. A Hybridization scheme of the SLA/PrV microarrays. Forty-eight hybridizations corresponding to 24 PrV/SLA slides were hybridized (two arrays per slide, see Methods). B Hybridization scheme of the Qiagen-NRSP8 microarrays. Thirty-two Qiagen-NRSP8 slides were hybridized. Each labeled target is represented by its dye Cy3 or Cy5. Each arrow linking Cy3 to Cy5 represents one hybridization on one array.

All time points (T0, T1, T2, T4, T8 and T12 h pi) for SLA/PrV hybridizations and only four time points for Qiagen-NRSP8 hybridizations (T1, T2, T4 and T8 h pi) were used. Twelve and eight different conditions were considered for the SLA/PrV and Qiagen-NRSP8 hybridizations, respectively. The hybridization scheme which can be defined as dye-switch was chosen to minimize the number of slides used and to test the impact of several factors on the results (conditions, labeling protocol, networks, day of experimentation and samples). A balanced loop design with two independent loops, each loop containing two replicates of PrV infection and mock-infection, was used (Figure 6). In total, 24 SLA/PrV slides and 32 Qiagen-NRSP8 slides were used in this experiment. All slides were processed in the same conditions. The slides were pre-soaked, prehybridized and hybridized with the same quantity of Cy3 and Cy5 labeled cDNA: 20 and 40 pmoles of each labeled cDNA for the SLA/PrV slides and for the Qiagen-NRSP8 slides, respectively (Pronto Kit, Corning, France). After hybridization for 16 hours at 42°C, the slides were washed according to a commercial protocol (Pronto kit, Corning, France) and dried by centrifugation (1500 rpm, 3 min).

SLA/PrV and Qiagen-NRSP8 slides were scanned on the Scanarray scanner (Perkin Elmer, France) and on the Microarrayreader scanner (Virtek, France), respectively. Signals were quantified with the Imagene version 5.1 software (Biodiscovery, USA). All the results were stored in the BioArray Software Environment (BASE) of SIGENAE (Système d'Information du projet d'Analyse des Genomes des Animaux d'Elevage) [20].

The SLA/PrV and the Qiagen-NRSP8 microarray data have been submitted to the GEO and received accession numbers GSE8676 and GSE9259, respectively.

### Statistical data analysis

The normalization and statistical analysis steps were performed with scripts written with R software. Functions contained in stats, anapuce and varmixt packages were used. Raw data were log2 transformed, normalized by lowess (f = 0.3) and the median of each block spotted on the slide was subtracted. Normalized data were analyzed with a linear model. For one gene and one target, the following linear model was used:

Y_cfnds _= μ_c _+ α_f _+ β_n _+ δ_d _+ ε_s _+ E_cfnds_

with Y_cfnds _= normalized intensity, μ_c _= condition effect (1 ≤ c ≤ o), α_f _= fluorochrome effect (1 ≤ f ≤ 2), β_n _= network effect (1 ≤ n ≤ q), δ_d _= effect of experiment day (1 ≤ d ≤ 2), ε_s _= sample effect (1 ≤ s ≤ r), E_cfnds _= random error, o = number of conditions, q = number of arrays, r = number of samples. Since the effect of samples was not significant (Student t test, p > 0.05), we did not keep it in the final linear model. Selected contrasts (defined by a linear combination of 2 or more factor level means with coefficients that sum to zero) were tested with the Student t test: infected condition against mock-infected condition at each time point. The differences observed for one time point were tested taking into account all the data for all the time points leading to a powerful statistical test. The false Discovery Rate (FDR) was calculated after the Student t test (R software, varmixt package). We selected differentially expressed genes with an FDR = 0.05.

Hierarchical clustering analysis (HCL, Eisen) was performed to analyze viral genes and cellular genes that were differentially expressed during infection (euclidian distance, average linkage) using TMeV software [53]. Averages of normalized intensities for all the mock-infected conditions and for each infected condition were calculated and these values were centered (median) by genes. The "k-means" method was used to identify groups of genes with similar expression kinetics (TMeV software, [53]). The optimal number of groups was determined by the "Figure of Merit" method (FOM).

### Functional analysis/Network and pathway analysis

The "Gene Ontology" annotations of differentially expressed cellular genes were obtained with the Panther software based on human orthologous genes [54]. The biological process sub-ontology was used to classify genes in different functional groups. The Ingenuity Pathways Analysis (IPA) program permitted the determination of the significant networks and the top functions associated with the differentially expressed genes at each time point (IPA 5.0, Ingenuity Systems Inc., USA; [55]). The IPA program searches the Ingenuity Pathway Knowledge Base (IPKB) for interactions (known from the literature) between the uploaded genes and all other genes contained in IPKB and generates a series of networks. The genes selected by the IPA network analysis are called focus genes. The Fisher exact test was used to assign statistical significance, and each network's score is displayed as the -log (P-value). Significant networks with a score greater than 3 were selected (p < 0.001) and top functions associated with these networks are presented (Table 4).

### Quantitative real time RT-PCR (qRT-PCR)

RNAs corresponding to mock-infected T0 and T1 and infected T0, T2, T4, T8, T12 samples from three replicates were used for real-time PCR analysis. Primers were chosen with the Primer express software or manually, one of each primer couple located on the exon-exon boundary (Table 7). Five differentially expressed porcine genes and two other porcine genes were chosen for real-time PCR testing. Each amplicon was sequenced and its sequence was aligned with the gene reference sequence. Two and a half μg of cleaned total RNA were reverse transcribed using Superscript II enzyme (Invitrogen, France) with Oligo(dT) primers (Invitrogen, France) and cDNAs were quantified with the 2100 Bioanalyzer (Agilent Technologies, France) and diluted to obtain a 30 ng/μl final concentration for all samples. Triplicate reactions were set up in a 20μl volume using 10 ng cDNA, PCR primers (300 nM final concentration) and SYBR Green PCR Master Mix (Applied Biosystem, USA). An ABI PRISM 7900 HT sequence detection system was used for monitoring the level of SYBR green fluorescence. A dissociation curve was produced at the end of the cycling phase to ensure a single PCR product and no primer dimer. Amplification efficiencies were determined for all genes by serial dilution of cDNA using E = 10^[-1/slope]^. We checked that a slope between -3.2 and -3.5 was obtained for each primer couple to calculate the relative expression levels of the selected genes by using the 2^-ΔΔCt ^method. The gene encoding Ribosomal Protein L32 (RPL32) was chosen as the internal reference for each sample. The mock-infected sample at T0 of each replicate was used as a calibrator for all samples of one replicate. The mean and the standard deviations for each time of each replicate were calculated.

**Table 7:** Sequence of primers for qRT-PCR.

Target	Oligo	Sequence	Size(bp)
RPL32	forward	TGCTCTCAGACCCCTTGTGAAG	22
	reverse	TTTCCGCCAGTTCCGCTTA	19
SLAIa	forward	CATCATTGTTGGCCTGGTTC	20
	reverse	CCTTTTTCACCTGAGCGC	18
TAP1	forward	CCAGTATCTCAGGGATGTTGCTG	23
	reverse	CGCTGCTTATAGCCCCACC	19
TAP2	forward	TGTTGGGTGAGACACTAATCCCTTA	25
	reverse	CAAAGGCATCAGGGTCAAAATC	22
PSMB9	forward	GCGCTTCACCACAAATGCTA	20
	reverse	TCCACACCAGCAGCTGTAATG	21
PSMB8	forward	TACCTGCTTGGCACCATGTCT	21
	reverse	AGTACAACCTGCACTCCTTGGC	22
PPIA	forward	GTCTCCTTCGAGCTGTTTGCA	21
	reverse	CCAAATCCTTTCTCCCCAGTG	21
TNF	forward	CCCAAGGACTCAGATCATCGTC	22
	reverse	AGCTGTCCCTCGGCTTTGA	19

### Flow cytometry

The anti-porcine MHC class I monoclonal antibody (mAb) PT85A and the anti-porcine MHC class II mAb MSA3 were purchased from VMRD (USA). The mAb HOPC-1 (IgG2a, Beckman Coulter, USA) was used as control Ab. PE-conjugated goat Abs to mouse IgG2a were purchased from Southern Biotech (USA).

PK15 cells from three kinetics replicates, either just after mock-infection or infection with the GFP-expressing PrV strain and 8 h after mock-infection or pi were trypsinized, resuspended in pig serum for 20 minutes at 4°C, washed in D-PBS and resuspended in FACS buffer. Cells were incubated with 50μl of diluted primary antibody (1/200 in FACS Buffer) for 30 minutes at 4°C and then, after washing, in 50 μl of diluted PE-conjugated goat anti-mouse IgG2a (Jackson ImmunoResearch Laboratories, 1/600 in FACS Buffer) for 30 minutes at 4°C. After two washes in FACS Buffer, the cells were fixed in BD CellFix (BD Biosciences, USA) solution (1/10 in sterile D-PBS) and analyzed using a FACScalibur flow cytometer (BD Biosciences, USA).

## Competing interests

The author(s) declare that they have no competing interests.

## Authors' contributions

LF produced the SLA/PrV microarray, participated in the choice of the experimental design, performed RNA extractions, hybridizations, data analysis, qRT-PCR experiments, flow cytometry experiments and drafted the manuscript.

CRG participated in the coordination of the study, the SLA/PrV microarray design, the choice of the experimental design and corrected the manuscript.

MC produced the viral amplicons spotted on the SLA/PrV microarray.

GL participated in the qRT-PCR experiments.

KH assisted the design and the spotting of the SLA/PrV slide.

PC participated in the design of the SLA/PrV slide.

SR elaborated the experimental design and helped with the data statistical analysis.

FL participated in the coordination of the study, the design of the viral amplicons, produced the purified PrV virions, infected the PK15 cells and corrected the manuscript.

All authors have read and approved the final manuscript.

## Supplementary Material

Additional file 1 List of differentially expressed cellular genes spotted onto the SLA/PrV microarray. Click here for file

Additional file 2 List of differentially expressed cellular genes spotted onto the Qiagen-NRSP8 microarray. Click here for file

Additional file 3 Oligonucleotides used for production of PrV amplicons. Click here for file

## References

[B1] Pomeranz LE, Reynolds AE, Hengartner CJ (2005). Molecular biology of pseudorabies virus: impact on neurovirology and veterinary medicine. Microbiol Mol Biol Rev.

[B2] Nauwynck H, Glorieux S, Favoreel H, Pensaert M (2007). Cell biological and molecular characteristics of pseudorabies virus infections in cell cultures and in pigs with emphasis on the respiratory tract. Vet Res.

[B3] Mettenleiter TC (2000). Aujeszky's disease (pseudorabies) virus: the virus and molecular pathogenesis--state of the art, June 1999. Vet Res.

[B4] Klupp BG, Hengartner CJ, Mettenleiter TC, Enquist LW (2004). Complete, annotated sequence of the pseudorabies virus genome. J Virol.

[B5] Ambagala AP, Solheim JC, Srikumaran S (2005). Viral interference with MHC class I antigen presentation pathway: the battle continues. Vet Immunol Immunopathol.

[B6] Mellencamp MW, O'Brien PC, Stevenson JR (1991). Pseudorabies virus-induced suppression of major histocompatibility complex class I antigen expression. J Virol.

[B7] Ambagala AP, Hinkley S, Srikumaran S (2000). An early pseudorabies virus protein down-regulates porcine MHC class I expression by inhibition of transporter associated with antigen processing (TAP). J Immunol.

[B8] Koppers-Lalic D, Rijsewijk FA, Verschuren SB, JA GVB, Neisig A, Ressing ME, Neefjes J, Wiertz EJ (2001). The UL41-encoded virion host shutoff (vhs) protein and vhs-independent mechanisms are responsible for down-regulation of MHC class I molecules by bovine herpesvirus 1. J Gen Virol.

[B9] Ambagala AP, Gopinath RS, Srikumaran S (2003). Inhibition of TAP by pseudorabies virus is independent of its vhs activity. Virus Res.

[B10] Jansen A, Yu J (2006). Differential gene expression of pathogens inside infected hosts. Curr Opin Microbiol.

[B11] Jenner RG, Young RA (2005). Insights into host responses against pathogens from transcriptional profiling. Nat Rev Microbiol.

[B12] Ray N, Enquist LW (2004). Transcriptional response of a common permissive cell type to infection by two diverse alphaherpesviruses. J Virol.

[B13] Brukman A, Enquist LW (2006). Suppression of the interferon-mediated innate immune response by pseudorabies virus. J Virol.

[B14] Blanchard Y, Le MN, Le CM, Blanchard P, Leger J, Jestin A (2006). Cellular gene expression survey of PseudoRabies Virus (PRV) infected Human Embryonic Kidney cells (HEK-293). Vet Res.

[B15] Tuggle CK, Wang Y, Couture O (2007). Advances in swine transcriptomics. Int J Biol Sci.

[B16] Renard C, Hart E, Sehra H, Beasley H, Coggill P, Howe K, Harrow J, Gilbert J, Sims S, Rogers J, Ando A, Shigenari A, Shiina T, Inoko H, Chardon P, Beck S (2006). The genomic sequence and analysis of the swine major histocompatibility complex. Genomics.

[B17] Zhao SH, Recknor J, Lunney JK, Nettleton D, Kuhar D, Orley S, Tuggle CK (2005). Validation of a first-generation long-oligonucleotide microarray for transcriptional profiling in the pig. Genomics.

[B18] Ledger TN, Pinton P, Bourges D, Roumi P, Salmon H, Oswald IP (2004). Development of a macroarray to specifically analyze immunological gene expression in swine. Clin Diagn Lab Immunol.

[B19] Bonnet A, Iannuccelli E, Hugot K, Benne F, Bonaldo MF, Soares MB, Hatey F, Tosser-Klopp G (2008). A pig multi-tissue normalised cDNA library: large-scale sequencing, cluster analysis and 9K micro-array resource generation. BMC Genomics.

[B20] (2007). Système d'Information du projet d'Analyse des GENomes des Animaux d'Elevage (SIGENAE). http://www.sigenae.org.

[B21] (2007). The Vertebrate Genome Annotation (VEGA) database. http://vega.sanger.ac.uk/index.html.

[B22] Bryant PA, Venter D, Robins-Browne R, Curtis N (2004). Chips with everything: DNA microarrays in infectious diseases. Lancet Infect Dis.

[B23] Hossain H, Tchatalbachev S, Chakraborty T (2006). Host gene expression profiling in pathogen-host interactions. Curr Opin Immunol.

[B24] Lovegrove FE, Pena-Castillo L, Mohammad N, Liles WC, Hughes TR, Kain KC (2006). Simultaneous host and parasite expression profiling identifies tissue-specific transcriptional programs associated with susceptibility or resistance to experimental cerebral malaria. BMC Genomics.

[B25] Zhang Y, Ohyashiki JH, Takaku T, Shimizu N, Ohyashiki K (2006). Transcriptional profiling of Epstein-Barr virus (EBV) genes and host cellular genes in nasal NK/T-cell lymphoma and chronic active EBV infection. Br J Cancer.

[B26] Zhu B, Ping G, Shinohara Y, Zhang Y, Baba Y (2005). Comparison of gene expression measurements from cDNA and 60-mer oligonucleotide microarrays. Genomics.

[B27] Wang HY, Malek RL, Kwitek AE, Greene AS, Luu TV, Behbahani B, Frank B, Quackenbush J, Lee NH (2003). Assessing unmodified 70-mer oligonucleotide probe performance on glass-slide microarrays. Genome Biol.

[B28] Stingley SW, Ramirez JJ, Aguilar SA, Simmen K, Sandri-Goldin RM, Ghazal P, Wagner EK (2000). Global analysis of herpes simplex virus type 1 transcription using an oligonucleotide-based DNA microarray. J Virol.

[B29] Kennedy PG, Grinfeld E, Craigon M, Vierlinger K, Roy D, Forster T, Ghazal P (2005). Transcriptomal analysis of varicella-zoster virus infection using long oligonucleotide-based microarrays. J Gen Virol.

[B30] Lin HW, Chang YY, Wong ML, Lin JW, Chang TJ (2004). Functional analysis of virion host shutoff protein of pseudorabies virus. Virology.

[B31] Lam Q, Smibert CA, Koop KE, Lavery C, Capone JP, Weinheimer SP, Smiley JR (1996). Herpes simplex virus VP16 rescues viral mRNA from destruction by the virion host shutoff function. EMBO J.

[B32] Abele R, Tampe R (2006). Modulation of the antigen transport machinery TAP by friends and enemies. FEBS Lett.

[B33] Wiertz EJ, Devlin R, Collins HL, Ressing ME (2007). Herpesvirus Interference with Major Histocompatibility Complex Class II-Restricted T-Cell Activation. J Virol.

[B34] Honda K, Takaoka A, Taniguchi T (2006). Type I interferon [corrected] gene induction by the interferon regulatory factor family of transcription factors. Immunity.

[B35] Ciccaglione AR, Stellacci E, Marcantonio C, Muto V, Equestre M, Marsili G, Rapicetta M, Battistini A (2007). Repression of interferon regulatory factor 1 by hepatitis C virus core protein results in inhibition of antiviral and immunomodulatory genes. J Virol.

[B36] Lundberg P, Welander PV, Edwards CK, van RN, Cantin E (2007). Tumor necrosis factor (TNF) protects resistant C57BL/6 mice against herpes simplex virus-induced encephalitis independently of signaling via TNF receptor 1 or 2. J Virol.

[B37] Andreeva L, Heads R, Green CJ (1999). Cyclophilins and their possible role in the stress response. Int J Exp Pathol.

[B38] Braaten D, Luban J (2001). Cyclophilin A regulates HIV-1 infectivity, as demonstrated by gene targeting in human T cells. EMBO J.

[B39] Geenen K, Favoreel HW, Olsen L, Enquist LW, Nauwynck HJ (2005). The pseudorabies virus US3 protein kinase possesses anti-apoptotic activity that protects cells from apoptosis during infection and after treatment with sorbitol or staurosporine. Virology.

[B40] Stevens JG, Kado-Boll GJ, Haven CB (1969). Changes in nuclear basic proteins during pseudorabies virus infection. J Virol.

[B41] Poon AP, Gu H, Roizman B (2006). ICP0 and the US3 protein kinase of herpes simplex virus 1 independently block histone deacetylation to enable gene expression. Proc Natl Acad Sci U S A.

[B42] Feierbach B, Piccinotti S, Bisher M, Denk W, Enquist LW (2006). Alpha-herpesvirus infection induces the formation of nuclear actin filaments. PLoS Pathog.

[B43] Pederson T, Aebi U (2005). Nuclear actin extends, with no contraction in sight. Mol Biol Cell.

[B44] Favoreel HW, Van MG, Adriaensen D, Nauwynck HJ (2005). Cytoskeletal rearrangements and cell extensions induced by the US3 kinase of an alphaherpesvirus are associated with enhanced spread. Proc Natl Acad Sci U S A.

[B45] Karger A, Schmidt J, Mettenleiter TC (1998). Infectivity of a pseudorabies virus mutant lacking attachment glycoproteins C and D. J Virol.

[B46] Riteau B, de VC, Lefevre F (2006). Trypsin increases pseudorabies virus production through activation of the ERK signalling pathway. J Gen Virol.

[B47] (2007). University of California Santa Cruz (UCSC) Genome Bioinformatics. http://genome.ucsc.edu/.

[B48] (2007). Interface de Cartographie Comparée pour l'Agronomie et pour la Recherche sur l'Evolution (Iccare) web server. http://bioinfo.genopole-toulouse.prd.fr/iccare/.

[B49] Muller C, Denis M, Gentzbittel L, Faraut T (2004). The Iccare web server: an attempt to merge sequence and mapping information for plant and animal species. Nucleic Acids Res.

[B50] (2007). Primer3. http://frodo.wi.mit.edu/cgi-bin/primer3/primer3_www.cgi.

[B51] (2007). The National Center for Biotechnology Information (NCBI). http://www.ncbi.nlm.nih.gov/.

[B52] (2007). The Gene Expression Omnibus (GEO). http://www.ncbi.nlm.nih.gov/geo/.

[B53] (2007). TIGR MultiExperiment Viewer (TMeV). http://www.tm4.org/mev.html.

[B54] (2007). Panther Classification System. http://www.pantherdb.org/.

[B55] (2007). Ingenuity Pathways Analysis (IPA). http://www.ingenuity.com.

