# Supplementary material for: Transcriptomic analysis of the dialogue between Pseudorabies virus and porcine epithelial cells during infection
Source: BMC Genomics. 2008 Mar 10;9:123. doi: 10.1186/1471-2164-9-123 (PMC2335119; doi:10.1186/1471-2164-9-123)
Supplement: Additional file 1 — List of differentially expressed cellular genes spotted onto the SLA/PrV microarray. [file 1471-2164-9-123-S1.doc]

Additional file 1

| Location | Clone name | Gene name | fold change | | | | | |
| --- | --- | --- | --- | --- | --- | --- | --- | --- |
|  |  |  | T0 | T1 | T2 | T4 | T8 | T12 |
| **SLA** | **Down-regulated** |  |  |  |  |  |  |  |
|  | SCAA0011.D.05 | EFHC1 | - | - | - | - | -3.9 | - |
|  | SCAA0035.B.05 | LY6G6C | - | - | - | - | -4.1 | - |
|  | SCAA0099.O.04 | SLA-7 | - | - | - | -3.5 | -3.6 | - |
|  | SCAA0122.E.09 | HIST1H2AL | - | - | - | -3.9 | -6.0 | -3.7 |
|  | SCAB0037.N.21 | CPNE5 | - | - | - | - | - | -4.3 |
|  | SCAB0046.B.09 | PI16 | - | - | - | -3.4 | -3.8 | - |
|  | SCAB0055.H.08 | SLC39A7 | - | - | - | - | -4.2 | - |
|  | SCAB0057.M.21 | HIST1H4J | - | - | - | -5.1 | -4.4 | -4.0 |
|  | SCAB0066.H.15 | KCTD20 | - | - | - | - | -4.4 | -6.2 |
|  | SCAB0073.K.18 | HIST1H2BK | - | - | - | -3.9 | -6.8 | -4.4 |
|  | SCAB0089.N.06 | C6orf89 | - | - | - | - | -4.1 | -6.1 |
|  | SCAB0103.A.08 | C6Orf49 | - | - | - | - | -4.2 | -3.7 |
|  | SCAB0107.H.22 | FKBP5 | - | - | - | -4.7 | -3.6 | - |
|  | SCAB0137.B.15 | HLA-DOB | - | - | - | -4.0 | - | - |
|  | SCAB0139.D.06 | RGL2 | - | - | - | - | - | -3.5 |
|  | SCAB0143.E.10 | ZNF193 | - | - | - | - | -4.0 | -5.3 |
|  | SCAC0044.H.08 | HLA-DMB | - | - | - | -4.6 | - | - |
|  | SCAN0007.O.20 | SLA classe 1 | - | - | - | -4.9 | -4.6 | -4.5 |
|  | SCAN0010.J.21 | SLA classe 1 | - | - | - | - | - | -5.9 |
|  | SCAN0029.D.12 | BTNL5/BTNL6 | - | - | - | - | -3.5 | -6.1 |
|  | SCAN0029.J.14 | ZFP57 | - | - | - | - | - | -5.2 |
|  | SCAN0032.G.07 | SLA classe 1 | - | - | - | - | -4.0 | - |
|  | SPAA0001.C.06 | SRF | - | - | - | - | -3.8 | - |
|  | SPAA0001.G.04 | C7H6orf48 | - | - | - | -3.5 | - | - |
|  |  |  |  |  |  |  |  |  |
|  | **Up-regulated** |  |  |  |  |  |  |  |
|  | SCAA0087.F.19 | SRPK1 | - | - | - | - | 5.9 | 7.3 |
|  | SCAB0026.I.21 | RPL10A | - | - | - | 3.6 | - | - |
|  | SCAC0037.O.09 | TAP1 | - | - | - | - | 3.9 | - |
|  | SPAA0001.E.01 | APOBEC2 | - | - | - | - | 4.8 | - |
|  | SPAA0001.F.04 | unknown | - | - | - | - | - | 3.6 |
|  | SPAB0001.B.03 | TRIM26 | - | - | - | - | - | 3.7 |
| **other location** | **Down-regulated** |  |  |  |  |  |  |  |
|  | SCAA0005.L.19 | CCT7 | - | - | - | - | - | -4.3 |
|  | SCAA0015.B.22 | PPIA | - | - | - | -3.9 | -4.8 | -4.8 |
|  | SCAA0024.I.14 | GSTZ1 | - | - | - | - | - | -3.8 |
|  | SCAA0084.B.07 | HM13 | - | - | - | -4.2 | -3.6 | - |
|  | SCAA0092.P.14 | NKAP | - | - | - | - | -3.8 | -5.0 |
|  | SCAA0095.M.15 | MYL9 | - | - | - | - | -4.4 | - |
|  | SCAA0108.M.01 | ZNF546 | - | - | - | -3.5 | - | - |
|  | SCAA0123.I.24 | RAN | - | - | - | -6.3 | -7.6 | -10.0 |
|  | SCAA0126.P.18 | KLF9 | - | - | - | - | -4.3 | -3.8 |
|  | SCAB0015.G.08 | HIST3H2BB | - | - | - | - | -4.2 | -5.4 |
|  | SCAB0044.D.01 | PFDN4 | - | - | - | - | -3.8 | - |
|  | SCAB0049.F.12 | unknown | - | - | - | - | -6.2 | -7.0 |
|  | SCAB0051.D.24 | H2AFJ | - | - | - | -5.5 | -5.1 | -3.7 |
|  | SCAB0137.A.14 | unknown | - | - | - | - | -4.3 | - |
|  | SCAC0027.C.07 | unknown | - | - | - | - | -4.5 | -6.7 |
|  | SCAC0038.L.19 | HSPA8 | - | - | - | - | -6.3 | -9.8 |
|  | SCAC0039.O.09 | unknown | - | - | - | - | -4.3 | -6.7 |
|  | SCAG0001.A.02 | 6S ribosomal protein L19 | - | - | - | - | -5.0 | -7.1 |
|  | SCAG0001.B.08 | 4S ribosomal protein S3 | - | - | - | - | - | -7.6 |
|  | SCAG0001.D.10 | LMD1_HUMAN Leiomodin 1 ( muscle form) | - | - | - | - | -5.6 | -7.2 |
|  | SCAG0001.F.06 | 4S ribosomal protein S3 | - | - | - | - | - | -7.4 |
|  | SCAG0001.G.03 | 834859.1.ss.5 | - | - | - | - | -3.8 | - |
|  | SCAG0001.H.12 | 5195573.1.ss.5 | - | - | - | - | -3.7 | - |
|  | SCAG0002.C.09 | scag0002c.c.09_3.1.ss.5 | - | - | - | - | - | -4.0 |
|  | SCAG0002.D.03 | SPG7_HUMAN Paraplegin (EC 3.4.24.-) | - | - | - | - | -3.6 | -4.8 |
|  | SCAG0002.D.05 | scag0002c.d.05_3.1.ss.5 | - | - | - | - | -3.9 | -4.4 |
|  | SCAG0002.D.07 | 6939895.1.ss.5 | - | - | - | - | - | -4.1 |
|  | SCAG0002.F.10 | Mitogen-activated protein kinase kinase kinase 3 | - | - | - | -4.0 | -5.1 | - |
|  | SCAG0002.G.03 | Apolipoprotein A-IV precursor | - | - | - | - | - | -3.6 |
|  | SCAG0002.G.08 | scag0002c.g.08_3.1.ss.5 | - | - | - | -4.5 | -6.6 | -8.0 |
|  | SCAG0002.G.10 | 4S ribosomal protein S3 | - | - | - | - | - | -6.0 |
|  | SCAG0002.H.09 | CWFN_SCHPO Cell cycle control protein cwf23. | - | - | - | -3.5 | - | - |
|  | SCAG0003.B.03 | unknown | - | - | - | - | - | -5.4 |
|  | SCAG0003.B.04 | COX3_PIG Cytochrome c oxidase polypeptide III . | - | - | - | - | - | -5.1 |
|  | SCAG0003.D.03 | SPMI_PIG Seminal plasma sperm motility inhibitor precursor. | - | - | - | - | - | -3.6 |
|  | SCAG0003.D.05 | Nucleobindin 2 precursor | - | - | - | - | - | -3.9 |
|  | SCAG0003.E.01 | Ubiquitin | - | - | - | - | -3.7 | -5.2 |
|  | SCAG0003.F.06 | scag0003c.f.06_3.1.ss.5 | - | - | - | - | -3.5 | - |
|  | SCAG0003.F.11 | unknown | - | - | - | - | -4.2 | - |
|  | SCAG0003.H.03 | Protein C2orf178 | - | - | - | - | - | -3.6 |
|  | SCAG0003.H.05 | 6S ribosomal protein L13 | - | - | - | - | -3.6 | - |
|  | SCAG0004.B.07 | 6639091.1.ss.5 | - | - | - | - | - | -4.5 |
|  | SCAG0004.C.01 | unknown | - | - | - | - | -3.9 | - |
|  | SCAG0004.C.02 | Ferritin light chain | - | - | - | -6.4 | -13.1 | -16.8 |
|  | SCAG0004.C.09 | scag0004c.c.09_3.1.ss.5 | - | - | - | - | -5.4 | -5.4 |
|  | SCAG0004.D.01 | 6S ribosomal protein L19 | - | - | - | - | -6.8 | -7.8 |
|  | SCAG0004.E.11 | scag0004c.e.11_3.1.ss.5 | - | - | - | - | -3.5 | - |
|  | SCAG0004.G.04 | scag0004c.g.04_3.1.ss.5 | - | - | - | - | -4.5 | -7.1 |
|  | SCAG0004.G.08 | 6S ribosomal protein L11 | - | - | - | - | -4.9 | -6.0 |
|  | SCAG0004.H.05 | Eukaryotic initiation factor 4A-II | - | - | - | - | -3.5 | -7.4 |
|  | SCAG0005.A.09 | scag0005c.a.09_3.1.ss.5 | - | - | - | - | - | -4.3 |
|  | SCAG0005.C.01 | Calcyclin | - | - | - | - | -3.7 | -5.5 |
|  | SCAG0005.F.04 | Putative methyltransferase HUSSY-3 | - | - | - | - | -3.8 | -5.0 |
|  | SCAG0005.F.06 | 2474809.1.ss.5 | - | - | - | - | -7.0 | -6.4 |
|  | SCAG0005.G.08 | scag0005c.g.08_3.1.ss.5 | - | - | - | - | -6.1 | -3.8 |
|  | SCAG0005.G.12 | Calmodulin | - | - | - | -4.0 | -6.5 | -7.7 |
|  | SCAG0005.H.04 | unknown | - | - | - | - | - | -3.5 |
|  | SCAG0005.H.07 | COX3_PIG Cytochrome c oxidase polypeptide III | - | - | - | - | - | -4.6 |
|  | SCAG0006.A.12 | unknown | - | - | - | -3.5 | - | - |
|  | SCAG0006.B.01 | Heterogeneous nuclear ribonucleoprotein K | - | - | - | - | -3.9 | -4.9 |
|  | SCAG0006.B.10 | 6S ribosomal protein L18 | - | - | - | - | -3.6 | -4.8 |
|  | SCAG0006.D.05 | scag0006c.d.05_5.1.ss.5 | - | - | - | - | -4.2 | -6.7 |
|  | SCAG0006.D.09 | EGD2_YEAST EGD2 protein (GAL4 DNA-binding enhancer protein 2). | - | - | - | - | -3.8 | -7.1 |
|  | SCAG0006.E.10 | scag0006c.e.10_3.1.ss.5 | - | - | - | - | -7.2 | -9.8 |
|  | SCAG0006.F.11 | SET protein | - | - | - | - | - | -3.9 |
|  | SCAG0006.H.06 | 6S ribosomal protein L17 | - | - | - | - | - | -4.1 |
|  | SCAG0006.H.12 | 4S ribosomal protein S8 | - | - | - | - | -4.3 | -6.9 |
|  | SCAG0007.A.04 | 4554690.1.ss.5 | - | - | - | - | -4.2 | -5.3 |
|  | SCAG0007.B.10 | 2466198.1.ss.5 | - | - | - | -3.9 | - | - |
|  | SCAG0007.D.12 | 3726836.1.ss.5 | - | - | - | -3.6 | - | - |
|  | SCAG0007.F.10 | Pyruvate kinase isozymes M1/M2 | - | - | - | - | -4.9 | - |
|  | SCAG0007.G.11 | scag0007c.g.11_3.1.ss.5 | - | - | - | -4.2 | -5.5 | -6.9 |
|  | SCAG0008.A.07 | 6S ribosomal protein L11 | - | - | - | - | - | -4.3 |
|  | SCAG0008.C.01 | Fibrinogen alpha chain [Contains: Fibrinopeptide A] | - | - | - | - | -3.7 | - |
|  | SCAG0008.F.09 | scag0008c.f.09_3.1.ss.5 | - | - | - | - | - | -5.6 |
|  | SCAG0008.H.02 | scag0008c.h.02_3.1.ss.5 | - | - | - | - | -3.7 | - |
|  | SCAG0009.B.06 | unknown | - | - | - | - | - | -4.8 |
|  | SCAG0009.C.01 | 6S ribosomal protein L37a | - | - | - | -4.1 | -7.2 | -6.0 |
|  | SCAG0009.C.07 | Cyclin-dependent kinases regulatory subunit 2 | - | - | - | - | - | -3.8 |
|  | SCAG0009.D.01 | 13 kDa leucine-rich protein | - | - | - | - | -5.4 | -5.3 |
|  | SCAG0009.D.06 | Ubiquitin | - | - | - | - | -7.2 | -8.7 |
|  | SCAG0009.E.01 | C-X-C chemokine receptor type 6 | - | - | - | - | - | -3.9 |
|  | SCAG0009.E.02 | U4/U6 small nuclear ribonucleoprotein Prp3 | - | - | - | - | -3.5 | - |
|  | SCAG0009.E.03 | Fibronectin | - | - | - | -4.2 | -5.0 | -7.0 |
|  | SCAG0009.E.12 | YV28_LEPIN Hypothetical UPF0176 protein LA3128. | - | - | - | - | - | -3.6 |
|  | SCAG0009.F.10 | 834859.1.ss.5 | - | - | - | - | -3.8 | - |
|  | SCAG0009.G.01 | scag0009c.g.01_3.1.ss.5 | - | - | - | -3.5 | - | - |
|  | SCAG0009.G.08 | scag0009c.g.08_3.1.ss.5 | - | - | - | - | -5.5 | -5.6 |
|  | SCAG0010.A.09 | AS16_HUMAN Ankyrin repeat and SOCS box containing protein 16 (ASB-16). | - | - | - | - | -6.0 | -9.2 |
|  | SCAG0010.A.12 | COX2_PIG Cytochrome c oxidase polypeptide III | - | - | - | - | -4.4 | -8.4 |
|  | SCAG0010.B.04 | COX3_PIG Cytochrome c oxidase polypeptide III. | - | - | - | - | - | -14.9 |
|  | SCAG0010.C.07 | 343888.1.ss.5 | - | - | - | - | - | -3.6 |
|  | SCAG0010.D.01 | 3835609.1.ss.5 | - | - | - | - | - | -4.9 |
|  | SCAG0010.D.07 | NADH-ubiquinone oxidoreductase chain 5 | - | - | - | - | -4.5 | -10.0 |
|  | SCAG0010.H.07 | 4S ribosomal protein S17 | - | - | - | - | -5.8 | -8.0 |
|  | SCAG0011.A.11 | Guanine nucleotide-binding protein G | - | - | - | - | -3.9 | - |
|  | SCAG0011.A.12 | scag0011c.a.12_3.1.ss.5 | - | - | - | -5.3 | -4.7 | -7.8 |
|  | SCAG0011.B.04 | unknown | - | - | - | - | -4.4 | - |
|  | SCAG0011.B.12 | scag0011c.b.12_5.1.ss.5 | - | - | - | - | - | -4.4 |
|  | SCAG0011.D.02 | Splicing factor 3B subunit 1 | - | - | - | -4.2 | -4.7 | - |
|  | SCAG0011.D.04 | unknown | - | - | - | - | - | -3.7 |
|  | SCAG0011.E.02 | scag0011c.e.02_3.1.ss.5 | - | - | - | - | -4.0 | - |
|  | SCAG0011.H.12 | unknown | - | - | - | - | -3.5 | - |
|  | SCAU0001.A.02 | ACTB | - | - | - | - | - | -7.4 |
|  | SCAU0001.B.05 | PPIA | - | - | - | -3.6 | -4.3 | -4.7 |
|  |  |  |  |  |  |  |  |  |
|  | **Up-regulated** |  |  |  |  |  |  |  |
|  | SCAG0001.A.04 | NifU-like protein | - | - | 0.0 | - | - | - |
|  | SCAG0001.E.10 | Calpain 2 large [catalytic] subunit precursor | - | - | - | 3.9 | 4.2 | 3.7 |
|  | SCAG0002.G.11 | Adiponutrin | - | - | - | 3.4 | - | - |
|  | SCAG0002.H.11 | RS11_HUMAN 40S ribosomal protein S11. | 3.5 | - | - | - | - | - |
|  | SCAG0003.B.02 | Forkhead box protein O1A | - | - | - | - | 7.0 | 6.6 |
|  | SCAG0003.E.11 | Tumor suppressor p53-binding protein 1 | - | - | - | - | 3.5 | 3.6 |
|  | SCAG0004.C.11 | Tumor protein p73 | - | - | - | - | 3.7 | - |
|  | SCAG0004.E.07 | M3KC_MOUSE Mitogen-activated protein kinase kinase kinase 12 (EC 2.7.1.37) | - | - | - | - | - | 3.9 |
|  | SCAG0005.C.04 | CED2_HUMAN Centaurin delta 2 (Cnt-d2). | - | - | - | - | 3.7 | - |
|  | SCAG0005.G.03 | SUF1_HUMAN Smad ubiquitination regulatory factor 1 | - | - | 0.0 | - | - | - |
|  | SCAG0006.B.05 | DNA-binding protein inhibitor ID-3 | - | - | - | 4.6 | 7.0 | 7.1 |
|  | SCAG0006.E.11 | scag0006c.e.11_3.1.ss.5 | - | - | - | - | 3.7 | - |
|  | SCAG0006.H.09 | 8976484.1.ss.5 | - | - | - | - | 3.7 | - |
|  | SCAG0007.C.06 | unknown | - | - | - | - | 3.5 | - |
|  | SCAG0007.D.09 | Epithelial protein lost in neoplasm | - | - | - | - | - | 3.5 |
|  | SCAG0007.G.02 | Pantothenate kinase 4 | - | - | - | - | - | 5.1 |
|  | SCAG0007.H.03 | unknown | - | - | - | - | 3.7 | - |
|  | SCAG0008.D.01 | SPBP_RAT Prostatic spermine-binding protein precursor | - | - | - | - | 4.2 | 6.4 |
|  | SCAG0008.E.12 | Zinc phosphodiesterase ELAC protein 2 | - | - | - | - | 3.7 | - |
|  | SCAG0009.B.05 | Jerky protein | - | - | - | - | 4.9 | 3.7 |
|  | SCAG0009.E.08 | Guanine nucleotide exchange factor DBS | - | - | - | 3.6 | - | - |
|  | SCAG0010.D.10 | Epithelial protein lost in neoplasm | - | - | - | - | - | 3.9 |
|  | SCAG0010.H.09 | 3847078.1.ss.5 | - | - | - | - | 4.6 | 4.2 |
|  | SCAG0010.H.12 | Transcription factor GATA-4 | - | - | - | - | 3.8 | - |
|  | SCAU0001.A.05 | CCR7 | - | - | - | - | 4.5 | - |

- gene not found differentially expressed between I and MI cells
