# Supplementary material for: Transcriptomic analysis of the dialogue between Pseudorabies virus and porcine epithelial cells during infection
Source: BMC Genomics. 2008 Mar 10;9:123. doi: 10.1186/1471-2164-9-123 (PMC2335119; doi:10.1186/1471-2164-9-123)
Supplement: Additional file 3 — Oligonucleotides used for production of PrV amplicons. [file 1471-2164-9-123-S3.doc]

Additional file 3

| amplicon name | transcript name | Sequence oligo sense | Sequence oligo antisense | Amplicon size (bp) |
| --- | --- | --- | --- | --- |
| ORF1 | ORF1 | TCCACAACGAGCCCGCGGGG | CGGGCGGTCTTCGGGGTAGC | 517 |
| UL54 | UL52/UL53/UL54 | GCCGACCTGCACCGCCTGTT | CCGTGCATGTACACGGGGACGA | 539 |
| UL53 | UL52/UL53 | GGGAGGCCTTCAACGCCACG | GCGGGCGTACAGCGGGAAGA | 521 |
| UL52 | UL52 | AGCAGGCCGTGCTGCTGGAG | CCAGTCGGGCTTGGCCACCA | 506 |
| UL51 | UL51 | ACGCTGGCGCGCACCTACCA | GCCAGCCTCCGGGCCTTCTC | 509 |
| UL50 | UL50 | CGCCACGGACGTGCCCTTCT | CGCTGCTCGGGGCGACAAAG | 422 |
| UL49.5 | UL49.5 | AGCCCACGGCCGCCGCCG | GGGGTCGGGCGCGTGCGG | 151 |
| UL49 | UL49/UL49.5 | CGGCTACGATGGCTACGGCTCCT | GGCCGCCTCGATCAGGTCCA | 502 |
| UL48 | UL48 | ACGGCGTGGTGCTGCTGTCG | GCGTCGCGGGGGATGAGGT | 502 |
| UL47 | UL47/UL48 | TCGGACATCCCCGACGACGA | CGCGATGACGAGCCGCTTGC | 507 |
| UL46 | UL46/UL47/UL48 | AGGGGGAGGGCGACGACGAG | CGGTGGCCGGTGAGGTCGAT | 526 |
| GII | UL28/UL27 | GCGCGGCATCGCCAACTTCT | CGTCGGGGTCCTCGCTCTCG | 470 |
| ICP18.5 | UL28 | GCCTGTACGCGGACCGCCTCT | TACGCCGGCCGTCTGGAAGC | 503 |
| UL29 | UL29 | GCACGTGTGCCCCCTGATGG | CGGGGCGCCCATGTCAAACA | 523 |
| UL30 | UL30 | GCGTCATCAACGGCGGCAAG | CGACCACGTAGGCGGGGTCCT | 513 |
| UL31 | UL31/UL32 | CGCGATCGCTACGCGCCCTA | CCGAGCAGGCGGTCCAGCAC | 524 |
| UL32 | UL32 | CCGACCTCGCCATGCTGCTG | TCCACAGCTCCCCGCACACG | 573 |
| UL33 | UL33 | ATCTGCGCGCGGCGATCC | GCCCAGCTCCACCCCGTCAC | 308 |
| UL34 | UL33/UL34 | CCCCTACGTGCGCGTGCAGA | CCGCAGCCCCAGGAGGGTGT | 514 |
| UL35 | UL33/UL34/UL35 | CGGACGATCACCGCGCAGAC | GGGGGCGAGGGCAGAAGGTC | 280 |
| UL361 | UL36 | CGAGCGAGGGAGAGGCCGAGA | GCGCTGCCCTTGAGGGTCGT | 550 |
| UL362 | UL36 | GGCGCGGCTCATCATCGACA | CGCGGTAGGCGGCGTACTTGG | 539 |
| UL37 | UL37maj/UL37min | CTTCCGCGCCGAGGTCAACA | AGGTTCACGGCGGCCCAGTC | 510 |
| UL38 | UL38 | GGGCCACGTGACGCAAGACC | ACGGTGCCGAGGCGCACGTA | 522 |
| UL39 | UL39 | GCGCCTCAACGCCGCCATC | CGGTCCGCGCACATGTCGAT | 540 |
| UL40 | UL39/UL40 | CCATCCGCCGCAAGGTGGAG | GGTGCTGCGCCGCTCGAAAA | 509 |
| UL41 | UL41 | TGCACACGACGGACACGGACA | CGGGGGCGGGGGTATGTGTT | 539 |
| UL42 | UL42 | GCCCCCAGGTGGCCAAGCTC | CGCTTGGCGATGGGCGTGTA | 517 |
| UL43 | UL43 | CGTCACGTGCGCCACGAACA | GACGTGGTCCGCCTCGAGCA | 515 |
| GIIINIA3 | UL44 | CGCGCGTCTCCGTGGTGAAC | TGACCAGCACGATGGCCAGGA | 513 |
| ORF2NIA3 | UL24/UL25/UL26/UL26.5 | GCCGTGGTCTCGTCGCTCCA | CCGAGCTGGCGTCGATGGTG | 517 |
| UL26NIA3 | UL24/UL25/UL26 | GGCCCGCTGCCCATCAACAT | AGCGGTCGCGCAGCAGCAT | 511 |
| ORF1NIA3 | UL24/UL25 | CGAGGACGCGGCCCTGCT | ACTGCCCGAGGCGCTTGTCC | 512 |
| UL25NIA3 | UL24/UL25 | GAGGGCGGGCGCATCTCGTA | CCCGGGGAAGAGCTGCGTGA | 504 |
| UL24NIA3 | UL24 | GTGGGGCCGCACCTGTGCTT | GCGGCCCGGCGACGTACT | 310 |
| TKNIA3 | UL23 | GCGCTTCATCGTCGGGGACA | TGCCGGATGTGGTCGCCGTA | 564 |
| GHNIA3 | UL22 | GGGGCGCCAGCTTCGTGTTC | GATGGCCGCCCCCACGAC | 455 |
| UL21NIA3 | UL21 | GCGACGACGACGACGGAGGA | GTCGCCGAAGGCGGTCAGGA | 437 |
| UL20NIA3 | UL20 | CGCGAAGCTGACGGGGGAGA | TGCATGATGGAGCGCGCGTA | 444 |
| UL191 | UL19maj/UL19min | CTCAGCGTGGACCGGCATCG | TGGGCCATCTCGACCCACAGG | 524 |
| UL192 | UL19maj/UL19min | ACTTTCGGCGCGCGTGCAAC | CGCTCGCGTGCTGGAAGCAG | 498 |
| UL18 | UL19/Ul19min | CCCAGGCCCGCGAGCTGAC | TCCACCACCGCGGCCTTCTC | 493 |
| UL15EX2 | UL15 | GTGCGCGTGGCCGTCGAG | TCGGGCAGCGTGCTCAGGTG | 443 |
| UL17 | UL17 | GCGCGCCGCCACTACCAGAC | CGCGGCGGCCGTCATCAG | 501 |
| UL16 | UL16/Ul17 | CGGTGAGCCGCCTGGACCTG | CCGGCGTCGTTCACCACCAC | 505 |
| UL15EX12 | UL15 | GGAGGACGCCGCCGAGGAG | CGTCGGGGAAGGTGACGGTGA | 484 |
| UL14 | UL14 | GGTCCGCGTAGTAGCGCCGCGTCGC | GCGTGAACCGCCGGTGCCTGTCCAG | 332 |
| UL142 | UL14 | TCCGGGCCTTTATGGCGGCC | AGCGCGTCCTCCGCGGCGTC | 208 |
| UL13 | UL13/UL14 | GGAGGCGGAGGAGGCGTGAG | GAGCGGCGGTACGCGTCCAT | 464 |
| UL12 | UL12/UL13/UL14 | CGGTTCTGCGGCGAGGATGG | AGATGAGCGCCGCGTTGTCG | 477 |
| UL11 | UL11/UL12/UL13/UL14 | CGTGCCCGTGCTGCTGATCG | GCGAGGCTTCGCGGAGGT | 286 |
| UL10 | UL10 | GCCGTGGTCGGGCTGATGCT | CCCGGGGGCCAGCAGGTACT | 468 |
| UL92 | UL9 | GAGGTCATGTCCACGCTGGGGCAGC | ACGAGAAGACGCAGACGTTGTCCCC | 447 |
| UL8.5 | UL8.5/UL9 | CGCTGGCCCCCGACTTCAAC | CCCCGGCGTTCCATCAGCAG | 478 |
| UL8 | UL8/UL8.5/UL9 | CTACTGCGCGCTGGGCTTCC | CGCCCCAGAAGCCGTCCTTG | 503 |
| UL7 | UL6/UL7 | CCTTCCGCGGGTTCGCCTTC | ACACGGCCTCGGCCATCTCG | 531 |
| UL6 | UL6 | TGCTCTGGCAGCCGCTGTGG | GGCCCCCGTTGTCCGCCTTC | 501 |
| UL5 | UL5 | CTCGTCGCACCGCGAGGTCA | CGGGGCGCTGCAGGAAGTTG | 503 |
| UL4 | UL4/UL5 | TGGGCAGACCGTGTGCGAGT | GGTGACGGTGGTGGCGATGG | 433 |
| UL3.5 | UL1/UL2/UL3/UL3.5 | CCTCCCGCCGCGTCTCCA | CGGGAGGGTGGCCGTCGAG | 503 |
| UL3 | UL1/UL2/UL3 | GGTGCTCACCTTCAGCCATCC | CCCCTCCTCGCCGTCTTCCA | 410 |
| UL2 | UL1/UL2 | ATGAGGCGGCCCTCAAGGAG | AGGCGCGTCCAGGCGAACAC | 503 |
| UL1 | UL1 | GTCCGCGCCTCGATGCGCCCC | GCGGGTGATCCTCATCGTCCC | 374 |
| LLTE1 | LLT (1^st^ exon) | TCACAGCAGCCGCGGACGCTGC | TCCGTCTCTCCCGCCCGGTGTC | 136 |
| EP02 | EP0/LLT | GCGGGGTGCACACGGAGGAT | GAGCCGAAGTGGGGGCAGGA | 512 |
| EP01 | EP0/LLT | GGGGCCGGGCACCCGGTTAAAA | GTGAGTGGCAGTGACAGGCGCT | 164 |
| LLTI2 | LLT intron | CTGCATATCGCGCCGGCCCCTTCCG | CACGGTGGTGGTCAGTACAGGGAAC | 442 |
| LLTE21 | LLT (2^nd^ exon) | GGGACCACCGGGACCCTCGGGACCA | TTGGGGAGATGGAGGCGGCCATCTT | 379 |
| IEP2 | IE180 | CGCTCGGGCCCATCAAGGTG | TCACCCGATGGGAGAAGGAGGA | 507 |
| IEP1 | IE180 | GCCATGAGCCGCCGCTACGA | AGAGGGCCGCGGCGTAGGTC | 484 |
| Ba5 | IE180/ LLT (2^nd^ exon) | CGGGAGGGTGGCCGTCGAG | CGGGAGGGTGGCCGTCGAG | 520 |
| LLTE222 | LLT (2^nd^ exon) | GCTTCCGTGTGCACGTCATTTGC | GGCGTCGCTTCCCGGTTCGGGTC | 524 |
| RSP40 | US1 | GGCCCGCTCTTTGGGGAGGA | CCCTCCTCGCCCTCTCCGTCT | 509 |
| PKNIA3 | US3maj/US3min | CTCCGCGGGCTCGCCTACCT | GGCGGCGGACGGGATCAAAC | 508 |
| GXGG | US3maj/US3min/US4 | CGCTTCTTCGCCGCCTCCAC | CACGTGGCGGTAGCCGTCGT | 490 |
| GP50GD | US6 | GTTCGCCCGCGTGGACCAG | GAGCGGTGGCGCGAGACG | 486 |
| GP63GI | US6/US7 | GCGCCGGCTGCTGTTCGTCT | ACGATCGTGGGCCCCAGCAC | 498 |
| GIGE | US8 | CACCGCGGCCGAGTACGTCA | TCCTCGGGGCGCACGTACAG | 507 |
| 11K2 | US9/US8 | CATGGACACGTTTGACCCCAGCGCC | ACACGTGCCTGGCGACGATGCCCCC | 296 |
| 28KNIA3 | US2 | CGGCTCGCTGGCCCTGCT | GTCGCCCCGGGTCACGATCT | 508 |
